# Single Cell‐Pair Proteomics for Decoding Immune‐Cancer Cell Interactions

**DOI:** 10.1002/advs.202414769

**Published:** 2025-01-22

**Authors:** Qin‐Qin Xu, Yi‐Rong Jiang, Jian‐Bo Chen, Jie Wu, Yi‐Xue Chen, Qian‐Xi Fan, Hui‐Feng Wang, Yi Yang, Jian‐Zhang Pan, Qun Fang

**Affiliations:** ^1^ Institute of Microanalytical Systems Department of Chemistry Zhejiang University Hangzhou 310058 China; ^2^ Key Laboratory of Excited‐State Materials of Zhejiang Province Zhejiang University Hangzhou 310007 China; ^3^ Single‐cell Proteomics Research Center ZJU‐Hangzhou Global Scientific and Technological Innovation Center Hangzhou 311200 China; ^4^ Engineering Research Center of Functional Materials Intelligent Manufacturing of Zhejiang Province Hangzhou 311200 China; ^5^ Key Laboratory for Biomedical Engineering of Ministry of Education, Cancer Center Zhejiang University Hangzhou 310007 China

**Keywords:** immune‐cancer cell interaction, mass spectrometry, microfluidic analysis, single cell‐pair proteomics

## Abstract

The efficacy of cancer immunotherapy is significantly influenced by the heterogeneity of individual tumors and immune responses. To investigate this phenomenon, a microfluidic platform is constructed for profiling immune‐cancer cell interactions at the single‐cell proteomics level for the first time. Based on the platform, a comprehensive workflow is proposed for achieving accurate single‐cell pairing of an immune cell and a cancer cell with low cell damage and high success rate up to 95%, cell pair co‐culture, and real‐time microscopic monitoring of the cell‐pair interactions, cell pair retrieval, mass spectrometry‐based proteomic analysis of singe cell pairs, and decoupling of the proteomic information for each cell within the cell pair with the stable‐isotope labeling method. With the workflow, the interactions of single natural killer (NK) cells and single K562 tumor cells are investigated based on real‐time images and single cell‐pair proteomics. Notably, an identification depth of over 1000 protein groups in a single cell‐pair is achieved, leading to the discovery of sub‐clusters of NK cells with different functions and the identification of important biomarkers for cancer treatments. This demonstrates the unique capability of the present platform in providing substantial and comprehensive datasets for profiling immune‐cancer cell interactions, discovering heterogeneous immune responses, and predicting biomarkers in the study of cancer immunotherapy.

## Graphical abstract

A single cell‐pair manipulation and proteomics approach is developed and the immune‐cancer cell interaction study at the single‐cell proteomics level is achieved for the first time. This platform integrates cellular interaction fluorescence images with the current cutting‐edge technique of in‐depth single‐cell proteomics to explore the heterogeneity of immune responses from the perspective of the whole proteome of individual immune cell and tumor cell.

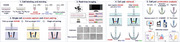

## 1 Introduction

In recent years, cancer immunotherapy (CIT) has demonstrated significant potential in the clinical treatment of cancer patients.^[^ ^1^ ^]^ However, its efficacy is influenced by the heterogeneity of individual tumors and immune responses.^[^ ^2^ ^]^ Thus, it is essential to characterize and understand the mechanism of immune‐cancer cell interactions to improve the effectiveness of CIT. Conventional experiments aimed at studying cellular interactions are typically conducted on bulk cells, providing an overview of the average behavior which may mask the inherent heterogeneity in immune responses due to the differences among individual cells at multiple molecular and phenotypic levels.^[^ ^3^ ^]^ To tackle this problem, it is crucial to study interactions between immune cells and tumor cells at the single‐cell resolution using single‐cell analytical technologies, which are expected to enhance the functional categorization of cell subpopulations and establish a link between single‐cell heterogeneity and treatment efficacy.^[^ ^4^ ^]^

In order to investigate intercellular interactions at the single‐cell level, several types of microfluidic devices have been developed, enabling flexible manipulation of single cells and precise control over the local microenvironment.^[^ ^5^, ^6^ ^]^ Single‐cell pairing is typically achieved using spatial confinements, such as microscale droplets,^[^ ^7^, ^8^ ^]^ microwells,^[^ ^9^, ^10^ ^]^ and hydrodynamic traps.^[^ ^11^, ^12^, ^13^ ^]^ In some cases, additional force fields, such as dielectrophoretic,^[^ ^14^, ^15^ ^]^ magnetic,^[^ ^16^ ^]^ optical tweezers,^[^ ^17^, ^18^ ^]^ acoustic trapping,^[^ ^19^ ^]^ or surface modification^[^ ^20^ ^]^ are used to further enhance the cell‐pairing success rates. Among these, the droplet‐based approach using continuously‐flowing micro‐volume droplets to encapsulate a single‐cell pair in single droplets has been widely used.^[^ ^21^ ^]^ However, the encapsulation of single cells in single droplets is usually a random process, and the success rates obey the Poisson distribution, resulting in the low cell‐pairing success rates. Therefore, some additional forces are needed to improve the performance, while this raises the risk of the negative impact of these forces on the viability of the captured cells.^[^ ^21^ ^]^ In addition, microwell chips mainly rely on gravity and cell size for cell capture, which are easy to fabricate and operate, but the cell‐pairing success rate of this approach is significantly affected by the sizes and shapes of the cells.^[^ ^22^ ^]^ The hydrodynamic trap chips rely on liquid flow and physical trapping structures to capture cells.^[^ ^23^ ^]^ Voldman et al. reported a 3‐step loading protocol and further designed a series of microfluidic deformability‐based cell pairing devices for cell pairing and fusion, providing high controllability and achieving cell‐pairing efficiencies between ≈40% and 80% using various cell types.^[^ ^24^, ^25^, ^26^ ^]^ However, it is inconvenient to extract specific cells from the closed microfluidic chips for subsequent research. Despite that many current approaches have achieved high throughput and high success rate of cell pairing, it remains a major challenge to develop a simple, easy‐to‐use and cell‐friendly single‐cell pairing approach that can not only capture and pair cells flexibly and precisely, but also recover the desired single‐cell pairs for downstream analysis to gain comprehensive label‐free protein information.

For comprehensive characterization of the immune responses, it is essential to use multiple analytical techniques to obtain multi‐dimensional signals and monitor complex behaviors during the intercellular interactions.^[^ ^27^, ^28^ ^]^ One of the most widely used techniques is microscopic imaging, which has been applied in observing various cellular behaviors, including immunological synapse formation,^[^ ^29^ ^]^ cytolytic activity,^[^ ^30^, ^31^ ^]^ and secretory activity,^[^ ^32^ ^]^ revealing the heterogeneity and correlation of these complex biological processes at the single‐cell level. In addition, the heterogeneity of single cells occurs at multiple molecular omics levels, and related studies would improve the understanding of the significant differences in functionality and behavior between individual cells.^[^ ^33^, ^34^, ^35^ ^]^ However, so far, there are few reports in the literature on acquiring and analyzing microscopic imaging information of interacting cell pairs in conjunction with their omics information, probably due to the difficulty in the accurate cell‐pair retrieval and subsequent omics analysis. Since proteins are major signaling molecules between cells, direct protein information is essential for assessing the functional state of immune cells and immune‐cancer interactions.^[^ ^36^ ^]^ Currently, the antibody‐based single‐cell protein analysis method has been successfully applied to analyze secretome‐mediated interactions between the paired single cells, enabling the identification of over a dozen secreted proteins produced during intercellular interactions.^[^ ^37^, ^38^, ^39^, ^40^ ^]^ To further enhance the depth of single‐cell proteomic identification, recently the mass spectrometry‐based single‐cell proteomics technique has been developed rapidly, which is capable of identifying 1000–2000 proteins in single tumor cells.^[^ ^41^, ^42^, ^43^, ^44^ ^]^ Such depths of identification have the potential to provide more information on intercellular interactions at the protein level.

Here, we constructed a microfluidic multifunctional platform to achieve the profiling of immune‐cancer cell interactions at the single‐cell proteomics level for the first time. The platform was composed of a single cell‐pair manipulation module, a cell‐pair co‐culture and microscopic monitoring module, and a single cell‐pair proteomic analysis module. Based on the platform, we developed the accurate single‐cell pairing, coculture, microscopic imaging, and retrieval approach, as well as the stable‐isotope labeling‐based single‐cell proteomic analysis approach, to address the challenges of studying the interactions of single immune cell and single tumor cell at the proteomics level. The platform and workflow were applied to investigate the interaction between natural killer (NK) cells and K562 tumor cells, and to obtain multi‐dimensional information to reveal the relationship between the heterogeneous immune responses and the corresponding protein expression profiles in the paired cells.

## 2 Results

### 2.1 Establishment of the Immune‐Cancer Interaction Profiling Workflow

The immune‐cancer cell interaction profiling platform consisted of three modules: a single cell‐pair manipulation module, a cell‐pair co‐culture and microscopic monitoring module, and a single cell‐pair proteomic analysis module.

**Figure 1:**
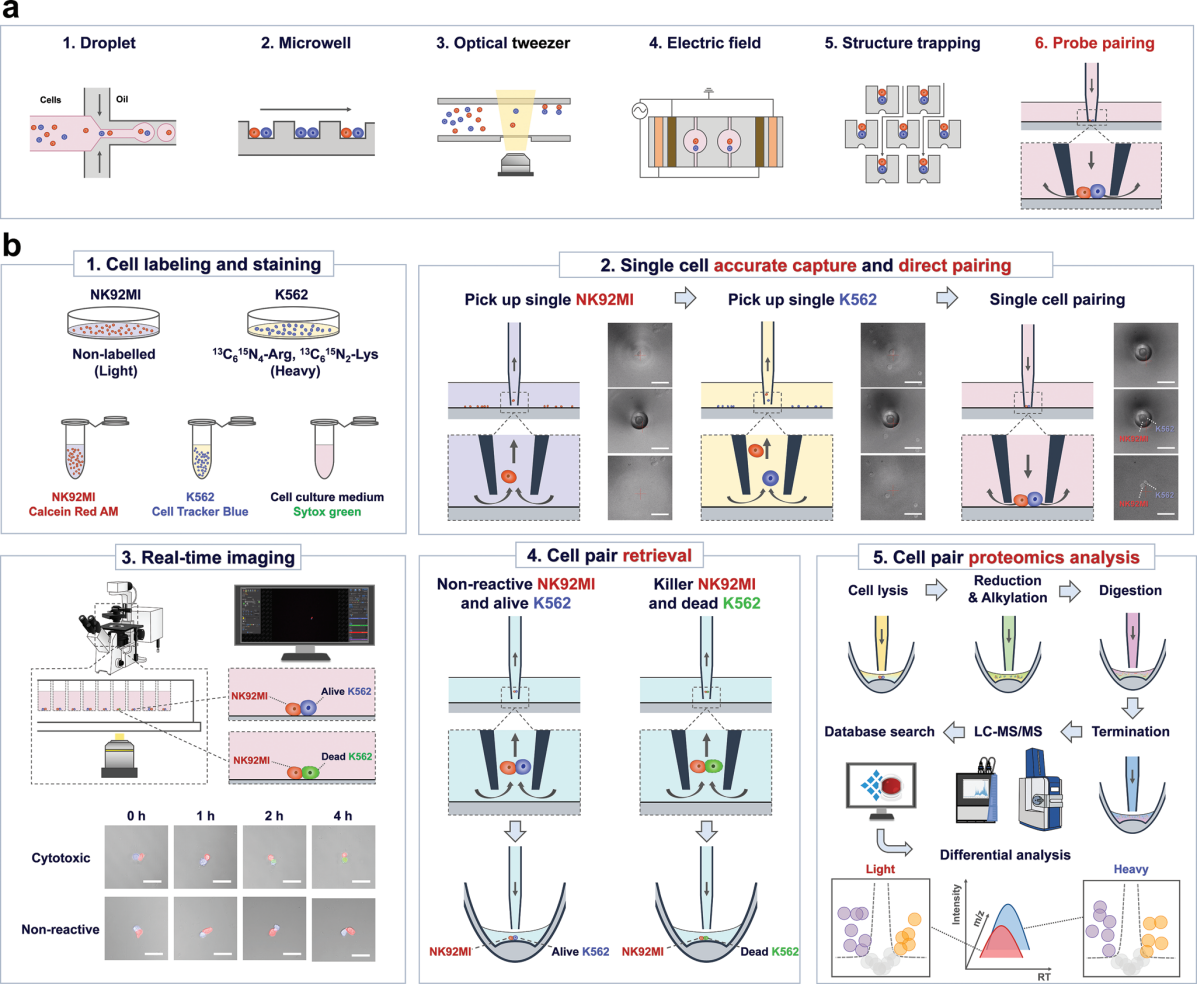
Schematic diagrams of different microfluidic single‐cell pairing approaches and the workflow for profiling the immune‐cancer cell interactions at the single‐cell proteomics level. a) Schematic diagrams of different types of microfluidic‐based strategies for single‐cell pairing, including (a1) droplet‐based microfluidic chip, (a2) microwell chip, (a3) optical tweezer, (a4) electric field, (a5) hydrodynamic trap chip, and (a6) capillary probe‐based manipulation used in this work; b) Schematic diagram of the workflow of the present platform for single immune‐cancer cell pairs analysis at the proteomics level. The workflow includes the steps of cell prelabeling and staining, accurate single‐cell capture and pairing, real‐time microscopic imaging, cell‐pair retrieval, and proteomic analysis. (b1) The NK92MI cells and K562 cells are cultured separately and respectively in regular media (Light) and media containing ^13^C_6_^15^N_2_ L‐lysine‐2HCl and ^13^C_6_^15^N_4_ L‐arginine‐HCl (Heavy). The NK92MI cells and K562 cells are stained respectively before single‐cell pairing. (b2) Schematic diagram and micrographs of the principle of single‐cell pairing procedure. The single cell‐pair manipulation module achieves nanoliter‐scale single‐cell pairing in three steps: sequential single‐cell capture, probe localization, and probe tip trapping of single cell pair; scale bar, 50 µm. (b3) Real‐time microscopic imaging of immune‐cancer interactions on‐chip for 4 h. The K562 cells (blue color) killed by the NK92MIs cell (red color) expressed the green fluorescence, otherwise remained blue; scale bar, 50 µm. (b4) The NK92MI‐K562 cell pair was washed in the PBS and transferred into an insert tube reactor by the probe. (b5) The proteome of the cell pair was analyzed using the shotgun‐based proteomics method including multi‐step pretreatment of cell lysis, protein reduction, alkylation, digestion, and LC‐MS/MS analysis, and the protein groups were identified by Spectronaut software. The individual proteomes of the single K562 cell and the single NK92MIs cell in the same cell pair were further analyzed separately based on their different protein labels.

The single cell‐pair manipulation module was developed based on the sequentially operated droplet array (SODA) technique previously developed by the authors’ group,^[^ ^45^, ^46^ ^]^ and mainly composed of an inverted fluorescence microscope and an automated microfluidic liquid handling robot with a capillary probe connected with a precise syringe pump. In studies of immune‐cancer cell interactions, direct contact between the paired immune and cancer cells is a key factor in the formation of the immunological synapses and the occurrence of cellular interactions.^[^ ^47^ ^]^ However, most of the existing methods, such as droplet‐based chips (**Figure** **1**a1) and microwell chips (Figure 1a2), could only confine two single cells to a specific area where direct contact between the paired cells is difficult to achieve. To address the challenge of direct contact between paired cells, the single cell‐pair manipulation module developed in this work used the tip of the capillary probe as a new type of spatial constraint device to overcome the limitations of the Poisson distribution method in single‐cell pairing. For achieving single‐cell pairing, we first used the capillary probe of the module to sequentially aspirate a single immune cell and a single tumor cell into the probe channel. Then, the relative position of the probe tip and the container for the single‐cell pair was precisely controlled using the module, to form a narrow gap smaller than the diameter of the two single cells between the probe tip end and the container surface. After that, the two single cells were gently dispensed to the surface of the container from the probe at a precisely controlled flow rate, during which the liquid flowed out of the narrow gap and the two cells were trapped inside the probe tip while located on the surface of the container. The inner diameter of the capillary probe tip had been pre‐selected to be less than the sum of the average diameters of the two cells, thereby the two cells trapped at the probe tip exhibited a state of mutual contact. After the capillary probe was removed from the container, both the single‐cell pairing and the direct contact of the two paired cells could be accomplished at the same time. With this approach, we could first image and evaluate the cell samples with bright field and fluorescence microscopy prior to cell capture and pairing, based on which select and determine the target cells to be captured and paired, and then carry out the actual one‐to‐one cell capture and pairing operation (Figure S1, Supporting Information). Compared with the previously‐reported cell capture and pairing approaches based on Poisson distribution or random capture, our approach has extremely‐strong directionality and definiteness in target cell capture and pairing, and can preserve the microscopic appearance and fluorescence properties of the target cells, which provides an additional informative dimension beyond the proteome. The difficulty in high probability of contact pairing of two cells with the random cell pairing approaches was also solved by our approach using the limiting effect of the capillary probe tip channel. As demonstrated by the subsequent experimental results, our approach enabled single cell accurate capture and direct pairing with a high pairing success rate up to 95% (Figure S2, Supporting Information). The single cell pairing process is schematically shown in Video S1 (Supporting Information).

Moreover, since only controllable relatively‐low flow rates were employed in the cell capture and pairing process, resulting fluid shear forces on the cells were similar to those experienced by cells in blood capillaries and had negligible effects on the cellular activity, which is different from the obvious effects of other external forces, such as optical tweezer (Figure 1a3) or electric field (Figure 1a4). Although the trap chips (Figure 1a5) could achieve direct cell pairing with minimized cell damage, accurate cell‐pair retrieval has always been a major challenge in various types of cell‐pairing microfluidic chip systems due to the obstructing effect of the closed microchannels on cell retrieval, thus limiting their ability to conduct further in‐depth analysis. In our platform (Figure 1a6), the capillary probe was coupled to the cell‐pair containers with open system structure, which significantly facilitated the cell recovery operation and the subsequent MS‐based proteomic analysis.

The cell‐pair co‐culture and microscopic monitoring module consisted of a live‐cell workstation and a confocal microscope. It has been widely recognized that immune‐cancer interaction is a dynamic and heterogeneous process, during which immune cells kill cancer cells at different moments and in different proportions.^[^ ^48^ ^]^ Real‐time monitoring of the status of immune cells and tumor cells is essential for accurate characterization of immune‐tumor cell interactions. Specific fluorescent labeling and high‐resolution imaging based on the confocal microscopy enabled accurate characterization of cell activity and apoptosis, while the coupled live‐cell workstation provided the necessary temperature and CO_2_ environment for cell survival in long‐time cell co‐culture and immune‐cancer interaction process.

Single‐cell proteomic analysis usually relies on a complex and multi‐step pretreatment process. Benefiting from the previous work of the authors’ group, the single cell‐pair proteomic analysis module was built based on the pick‐up single‐cell proteomic analysis (PiSPA) workflow to achieve nanoliter‐scale cell‐pair sample pretreatment,^[^ ^44^ ^]^ LC injection and separation, MS/MS detection and data analysis. However, the immune synapse formed during the immune‐cancer interaction is a zone where the membranes of both cells are apposed,^[^ ^49^ ^]^ making it difficult to separate the two single cells that are in contact and interacting with each other without damaging the cells. If the proteomic analysis is performed on the whole of a single cell‐pair, what is obtained will be the mixed proteomic information of the two interacted cells instead of their respective independent information, which will seriously affect the accuracy and depth of the interaction study. In order to obtain the independent proteomic information of the interacted single cells, we developed the stable isotope labeling‐based respective proteomics analysis approach for single cell‐pairs. Before the experiment, one of the interacted cells (e.g. tumor cells in this experiment) was isotopically labeled with ^13^C_6_^15^N_4_‐Arg and ^13^C_6_^15^N_2_‐Lys in cell culture process using the SILAC method;^[^ ^50^ ^]^ then the cell capture, pairing, interaction, and cell‐pair retrieval experiments were performed, followed by pretreatment, LC separation and MS detection of the single cell‐pair sample; finally, the respective proteomic information of the interacted individual cells were acquired by analyzing and decoupling the MS data.

Based on the platform, we established a five‐step workflow for profiling the immune‐cancer cell interactions at single‐cell proteomics level.

1. The tumor cells for interaction study were precultured with the medium containing two heavy‐isotope labeled amino acids, ^13^C_6_^15^N_4_‐Arg, and ^13^C_6_^15^N_2_‐Lys, while the immune cells were cultured under the non‐labeled routine conditions.
2. Before cell capture, both the suspensions of the tumor and immune cells were stained by a nucleic acid dye of SYTOX Green to distinguish the normal cells from dead cells. Then, under the bright‐field and fluorescence observation of the microscope, the target single tumor and immune cells with good viability were sequentially selected, captured, and directly paired using the single cell‐pair manipulation module.
3. The cell‐pairs were co‐cultured and the immune‐cancer interactions within the cell pairs were real‐time observed for 4 h with the cell‐pair co‐culture and microscopic monitoring module.
4. The cell pairs with different immune responses were retrieved by the single cell‐pair manipulation module.
5. The retrieved cell pairs were subjected to single cell‐pair proteomic analysis including multi‐steps of cell lysis, protein reduction, alkylation, digestion, reaction termination, and LC‐MS/MS analysis using the single cell‐pair proteomic analysis module.

The whole operation process is schematically shown in Figure 1b.

### 2.2 Evaluation and Performance of the Single Cell‐Pair Manipulation Module

**Figure 2:**
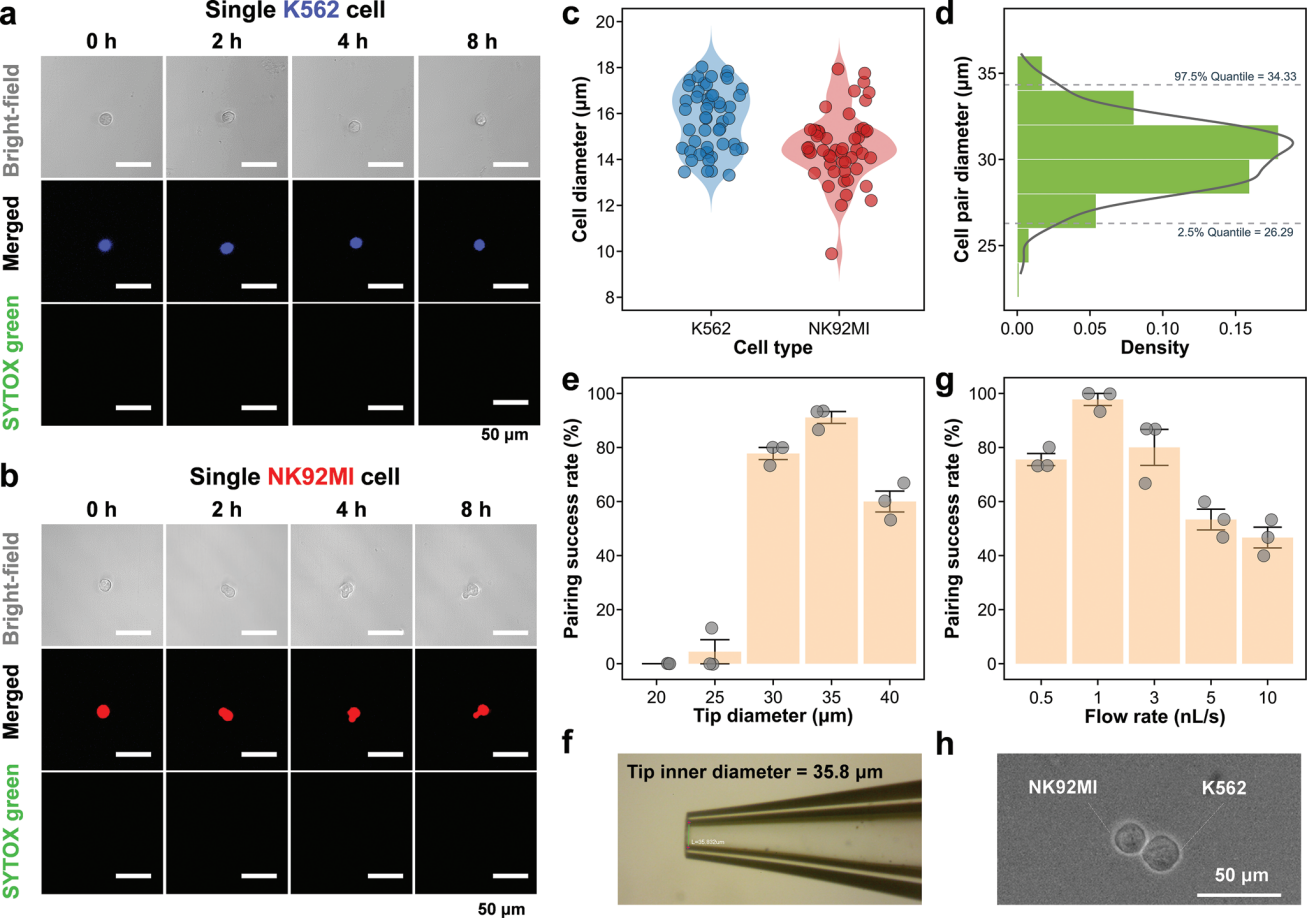
Evaluation and performance of the single cell‐pair manipulation module. a) A typical series of microscopic images of a single K562 cell after capture during an 8 h culture. The K562 cells were labeled with Cell Tracker Blue (10 µM). The single K562 cell was captured using a capillary probe with a 35 µm tip inner diameter at a flow rate of 1 nL/s, after which it was cultured in the NK92MI special medium containing 2.5 µM SYTOX green. We conducted the experiment using 16 single K562 cells, all of them remained viable (blue) during 8 h culture (n  =  16). b) A typical series of microscopic images of a single NK92MI cell after capture during an 8 h culture. The NK92MI cells were labeled with Calcein Red‐Orange (5 µM). The single NK92MI cell was captured using a capillary probe with a 35 µm tip inner diameter at a flow rate of 1 nL/s, after which it was cultured in the NK92MI special medium containing 2.5 µM SYTOX green. We conducted the experiment using 16 single NK92MI cells, all of which remained viable (blue) during 8 h culture (n  =  16). c) The measured diameters of single NK92MI cells (n = 50) and single K562 cells (n = 50). d) The frequency histogram with density curve of theoretical diameters of single NK92MI‐K562 cell pairs. e) The optimization of the tip inner diameter of the capillary probe for single‐cell pairing. The average single‐cell pairing success rates were 0%, 4%, 77%, 91%, and 60% (n  =  3) with the tip inner diameters of 20, 25, 30, 35, and 40 µm, respectively. The dispensing flow rate was 1 nL/s. f) Typical image of the tip of a capillary probe with a tip inner diameter of 36 µm for manipulation of single‐cell pairings. g) The optimization of the dispensing flow rate for single‐cell pairing. The average success rates for single‐cell pairing were 75%, 97%, 80%, 53%, and 46% (n  =  3) at the flow rates of 0.5, 1, 3, 5, and 10 nL s^−1^, respectively. The tip inner diameter of the probe was 35 µm. h) Typical image of a single NK92MI‐K562 cell pair.

During the process of single‐cell capture, a cell activity monitoring assay was conducted to evaluate the impact of the single cell‐pair operation on cell viability. In this assay, single K562 cells (**Figure** **2**a) and single NK92MI cells (Figure 2b) were captured and dispensed into wells of 384‐well plates containing the NK92MI special media at a dispensing flow rate of 1 nL/s, which was much lower than those of blood flow in normal human venae cavae^[^ ^51^ ^]^ and could be considered to be non‐damaging to the single cells. The NK92MI special media had been tested to have no obvious impact on the activity of the tumor cells. As shown in the results of live‐cell fluorescence imaging (Figure S3, Supporting Information), all single cells (n = 32) remained in good viability for 8 h, and even proliferation was observed in some cells, demonstrating that the fluid shear force from the capillary probe and the on‐chip culture conditions had negligible impact on the cellular activity. Thus, it could be surmised that the death of K562 cells observed in the interaction experiment of the single‐cell pairing was mainly caused by the immune‐cell‐mediated cytotoxicity by NK92MI cells.

We optimized various parameters of the single cell‐pair manipulation module that affected the performance of cell pairing. Before optimization, the diameters of 50 single NK92MI and K562 cells were measured, obtaining an average of 14.5 µm (n = 50) and 15.7 µm (n = 50), respectively (Figure 2c). Based on these data, the theoretical average diameter of cell pairs was estimated at approximately 30 µm (Figure 2d). Based on this theoretical average diameter, we chose to optimize the tip inner diameter of the capillary probe in the range of 20–40 µm, by using each probe to conduct 15 consecutive single‐cell pairing and calculating their pairing success rates. The experiment for each inner diameter condition was repeated three times with three capillary probes. Among them, the 35‐µm tip probes demonstrated the highest pairing success rates in the three replicate experiments, as 93%, 100%, and 100% (n = 15), respectively (Figure 2e). The results showed that a probe with a tip inner diameter larger than the diameter of the cell pairs by 5–10 µm could achieve a relatively higher success rate and stability for single‐cell pairing. The dispensing flow rate used in cell pairing was also optimized at flow rates of 0.5, 1, 3, 5, and 10 nL s^−1^, and 15 consecutive single‐cell pairing experiments were carried out at each flow rate using the probe with 35 µm tip inner diameter (Figure 2f). As shown in Figure 2g, the pairing success rate followed a bell‐shaped curve as the dispensing flow rate increased. Excessively high flow rates caused the cells to be squeezed out of the gap between the probe tip and the container surface, resulting in low pairing success rates and potential cell damage. Conversely, if the flow rate was too low, it might not provide sufficient driving force to allow the cells to close contact with each other, also leading to a decrease in the pairing success rate. Therefore, a capillary probe with a 35 µm tip inner diameter and a dispensing flow rate of 1 nL s^−1^ was chosen for single cell pairing, and an average pairing success rate of 98% was achieved. One representative image of the NK92MI‐K562 cell pair formed at the optimized conditions is shown in Figure 2h.

To further evaluate the universality of the single cell‐pair manipulation module, pairing experiments were conducted between NK92MI cells and A549 cells, as well as between NK92MI cells and Nalm‐6 cells with smaller cell size. The success rates for these pairing experiments were calculated to be 100% (15/15) and 80% (12/15) after 15 consecutive single‐cell pairings, respectively (Figure S4, Supporting Information). This shows that the single‐cell pairing approach is applicable to different types of cell pairing systems, including the pairing of suspension cells with adherent cells, and the pairing of suspension cells with different diameters, which indicates that this approach has broad application prospects.

### 2.3 Dynamic Monitoring of Immune‐Cancer Cell Interactions

It is widely recognized that the killing of tumor cells by NK cells is a dynamic and heterogeneous process, and not all NK‐cancer interactions result in target cell death.^[^ ^52^, ^53^ ^]^ Therefore, it is necessary to directly monitor this process in real time and in a controllable microenvironment.

To investigate the interactions of immune cells and tumor cells, at the preliminary stage of this work, we compared two modes: co‐culture of a large number of cells and single‐cell pairs, and observed that the killing rates of immune cells to tumor cells were similar in both modes, which were approximately 30% and 24%, respectively. However, when co‐culturing large numbers of cells, cell–cell interactions could not be precisely controlled, leading to uncertainty in the results. Under the co‐culture mode of a large number of cells, different pairs of cells may have different contact initiation times and durations, and the number of interacting cells is uncertain. In addition, there may be cross‐interference between different pairs of cells, making it difficult to assess the effect of single‐cell heterogeneity. In contrast, the co‐culture mode of single‐cell pairs allows the reliable control of the initial state and interaction conditions for each cell pair, the continuous monitoring of the interaction process, and the accurate investigation of the effect of single‐cell heterogeneity on the immune response.

In the present work, we used the fluorescence microscope and the combination of three kinds of dyes Calcein Red‐Orange AM, CellTracker Blue CMAC, and SYTOX Green to clearly differentiate the two cell types and evaluate their activity. The microscopic monitoring method was used to ensure the viability of the target both cells prior to cell capture and interaction. During the interaction, K562 cells transitioned from blue to green, indicating the cell death due to NK cell killing.

**Figure 3:**
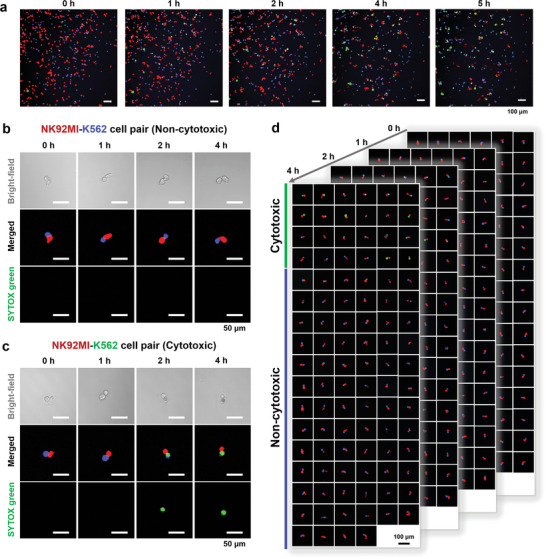
Real‐time viability tracking of NK92MI‐K562 cell interactions. a) A series of typical fluorescence images captured during the monitoring of the immune‐cancer interactions in a bulk mixed sample of NK92MI and K562 cells. More details are shown in Figure S5b (Supporting Information). Representative confocal images of the non‐cytotoxic dynamic process of a single NK92MI cell (red) against the paired single K562 cell (blue); scale bar, 50 µm. c) Representative confocal images of the cytotoxic dynamic process of a single NK92MI cell (red) against the paired single K562 cell (blue); scale bar, 50 µm. d) Confocal images of 24 NK92MI‐K562 cell pairs showing cytotoxic results and 76 NK92MI‐K562 cell pairs showing non‐cytotoxic results during 4 h co‐culture and interaction process; scale bar, 100 µm. More details are shown in Figure S6.

We first co‐cultured NK92MI cells and K562 cells with a cell number ratio of 1:1 and monitored the immune‐cancer interaction in the bulk sample. A total of 8 h of incubation was performed and the cells were monitored with florescence imaging at 5‐min intervals. As shown in **Figure** **3**a, ≈30% of the NK92MI cells exhibited cytotoxicity within 8 h, and most of the killing behavior occurred within the first 4 h (Video S2, Supporting Information). It has been reported that a large proportion of cytotoxic NK cells induced cytotoxicity within the first 4 h of interaction with K562 cells, and the number of cell‐killing events decreased over time during the interaction.^[^ ^7^, ^54^ ^]^ Therefore, the duration time of monitoring cellular interactions at the single‐cell level was chosen as 4 h, which allowed a significant proportion of NK cells to adequately exhibit their cytotoxicity heterogeneity.

The cytotoxicity of NK92MI cells against K562 cells at the single‐cell level was then assessed using the cell‐pair co‐culture and monitoring module. After the interaction between the K562 and NK92MI cells, a large proportion of single K562 cells in the cell pairs exhibited consistent blue fluorescence intensities, demonstrating sustained viability (Figure 3b). As shown in Figure 3c, the blue fluorescence intensities of a small number of K562 cells decreased due to membrane disruption and dye leakage, at the same time their green fluorescence intensities began to increase, which is the sign of cell death. Typical videos of the dynamic change of the cytotoxic and non‐cytotoxic cell pairs are shown in Video S3 (Supporting Information). We performed the interaction experiments of a total of 100 NK92MI‐K562 cell pair and their time‐lapse dynamic images were collected. Among them, 24 cell pairs exhibited cell killing behaviors with an overall killing rate of 24% (Figure 3d), showing the cytotoxic heterogeneity of the NK cells, which is consistent with the previously reported result.^[^ ^7^, ^26^ ^]^

### 2.4 SILAC‐Based Single Cell‐Pair Proteomic Analysis

**Figure 4:**
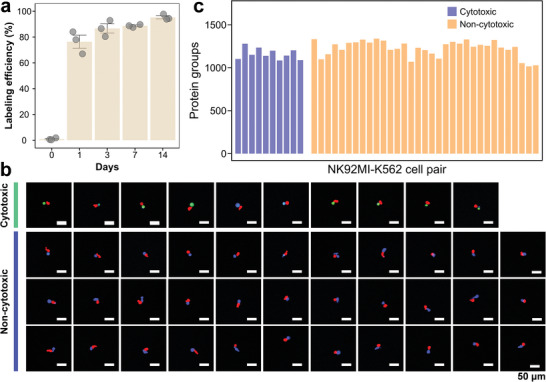
SILAC‐based single cell‐pair proteomic analysis. a) SILAC labeling efficiencies for single K562 cells in different labeling culture times of 0, 1, 3, 7, and 14 days. The labeling efficiency of single K562 cells gradually increased with increasing culture time, with an average of 95.3% after 14 days of culture (n = 3). b) Quantified protein group numbers of NK92MI and SILAC (heavy)‐labeled‐K562 cell pairs under the DIA mode. On average, 1163 ± 62 protein groups were quantified in the cytotoxic cell pairs (n = 10), and 1227 ± 91 protein groups were quantified in the non‐cytotoxic cell pairs (n = 33). c) Confocal images of 10 NK92MI‐SILAC (heavy)‐labeled‐K562 cell pairs showing cytotoxic results and 33 NK92MI‐SILAC (heavy)‐labeled‐K562 cell pairs showing non‐cytotoxic results during 4 h co‐culture and interaction process.

For single cell‐pair proteomic analysis, the SILAC‐based quantitative approach was used to achieve the simultaneous and respective single‐cell proteomic analysis for both the NK92MI cell and K562 cell in the cell pair, with which the NK92MI cells were non‐labeled (Light), while the K562 cells were labeled with ^13^C_6_^15^N_4_‐Arg and ^13^C_6_^15^N_2_‐Lys (Heavy). We tested the labeling efficiency of single K562 cells and an average efficiency of 95.3% (n = 3) was obtained after 14 days of culture in the isotope‐labeled medium (**Figure** **4**a). In the analysis of the MS data, the quantity of the heavy channel represents the proteins derived from the K562 cells, while the quantity of the light channel represents the proteins derived from the NK92MI cells, thus enabling the respective analysis of single NK92MI cells and single K562 cells of the cell pairs.

For the validation of this quantitative approach, we first evaluated the repeatability of the LC‐MS/MS method by running a quality control (QC) sample containing 400 pg of commercial HeLa cell digest. As shown in the Venn diagram (Figure S7a, Supporting Information), 92% of the total protein groups were quantified in all runs (n = 5). In the correlation analysis (n = 5), the correlation coefficients (CCs) of proteome expressions in the QC sample were all higher than 0.92 (Figure S7b, Supporting Information), indicating that this LC‐MS/MS method had good repeatability in proteome analysis. In addition, to evaluate the quantitative accuracy of the LC‐MS/MS method, a benchmark experiment was performed using the mixtures of digested HeLa and yeast samples in different proportions of 3:1, 2:2, and 1:3 (n = 3) with a total amount of proteins of 400 pg. The relative difference values between the medians of the protein abundance ratios and the theoretical calculated values ranged from 2.5% to 9.9% for the HeLa samples (Figure S8a, Supporting Information) and from 4.2% to 13.5% for the yeast samples (Figure S8b, Supporting Information), respectively, indicating that the present approach had an accurate quantification capability. The SILAC‐based cell‐pair proteomic analysis was conducted using 43 labeled immune‐tumor cell pairs (Figure 4b). Under the data‐independent acquisition (DIA) mode, an average of 1163 ± 62 protein groups were identified in 10 cytotoxic cell pairs, while an average of 1227 ± 91 protein groups were identified in 33 non‐cytotoxic cell pairs (Figure 4c).

**Figure 5:**
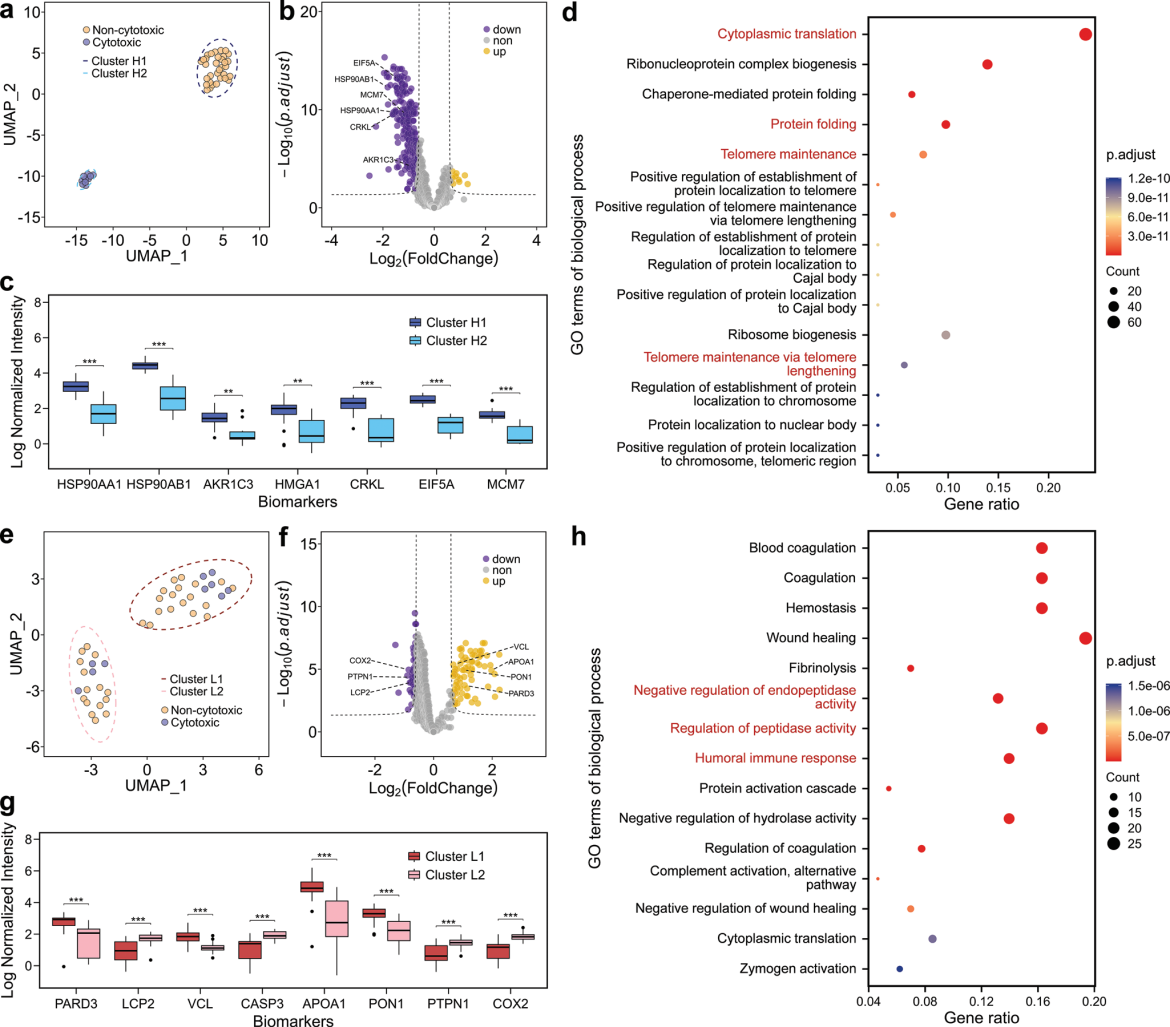
Bioinformatics analysis of NK92MI and SILAC (heavy)‐labeled‐K562 cell pairs. a) UMAP clustering analysis of the heavy‐labeled K562 cells in the cell pairs, with two clusters of cluster H1 (live K562 cells) and cluster H2 (dead K562 cells) aggregated out. b) Volcano plot showing 11 up‐regulated proteins and 257 down‐regulated proteins screened out in the heavy‐labeled K562 cells of cluster H2 compared to cluster H1. c) Comparisons of the quantitative expression levels of HSP90AA1, HSP90AB1, AKR1C3, HMGA1, CRKL, EIF5A and MCM7 in cell pairs of cluster H1 (n = 33) and cluster H2 (n = 10). ^∗∗∗^ *p* = 1.24e‐04 for HSP90AA1, ^∗∗∗^ *p* = 9.36e‐05 for HSP90AB1, ^∗∗^ *p* = 1.60e‐03 for AKR1C3, ^∗∗^ *p* = 2.14e‐03 for HMGA1, ^∗∗∗^ *p* = 4.51e‐05 for CRKL, ^∗∗∗^ *p* = 7.82e‐06 for EIF5A, and ^∗∗∗^ *p* = 8.17e‐05 for MCM7. d) Gene ontology (GO) term enrichment analysis (*p*_*adjusted*_ < 0.05 and q < 0.05) of the differential proteins between the heavy‐labeled K562 cells of cluster H1 and cluster H2. e) UMAP clustering analysis of the non‐labeled NK92MI cells of cell pairs, with two clusters of cluster L1 and cluster L2 aggregated out. f) Volcano plot showing 76 up‐regulated proteins and 53 down‐regulated proteins screened out in the non‐labeled NK92MI cells of cluster L1 compared to cluster L2. g) Comparisons of the quantitative expression levels of PARD3, LCP2, VCL, CASP3, APOA1, PON1, PTPN1 and COX2 in cell pairs of cluster L1 (n = 24) and cluster L2 (n = 19). ^∗∗∗^ *p* = 2.80e‐04 for PARD3, ^∗∗∗^ *p* = 3.33e‐05 for LCP2, ^∗∗∗^ *p* = 1.01e‐06 for VCL, ^∗∗∗^ *p* = 8.56e‐06 for CASP3, ^∗∗∗^ *p* = 3.33e‐05 for APOA1, ^∗∗∗^ *p* = 2.63e‐05 for PON1, ^∗∗∗^ *p* = 1.04e‐05 for PTPN1, and ^∗∗∗^ *p* = 2.27e‐06 for COX2. h) GO term enrichment analysis (*p_adjusted_* < 0.05 and q < 0.05) of the differential proteins between the non‐labeled NK92MI cells of cluster L1 and cluster L2. In c) and g), ^∗^ *p* < 0.05, ^∗∗^ *p* < 0.01, ^∗∗∗^ *p* < 0.001, Student's t test.

To understand the responses of the target cells (K562 cells) to effector cells (NK92MI cells), we analyzed the heavy channel data using UMAP clustering after data normalization and batch correction. The result showed that two groups were clearly clustered corresponding to the conditions of the K562 cells. Cluster H1 (n = 33) consisted of live K562 cells, while cluster H2 (n = 10) consisted of dead K562 cells (**Figure** **5**a). In comparison to cluster H1, a total of 268 protein groups were identified as proteins with differential abundances in cluster H2 (Figure 5b). Among them, some reported overexpressed proteins in chronic myelogenous leukemia (CML) or potential targets for cancer treatments were observed to exhibit downregulation in cluster H2 (Figure 5c), such as HSP90AA1^[^ ^55^ ^]^ and HSP90AB1^[^ ^56^ ^]^ of HSP90 family, AKR1C3,^[^ ^57^ ^]^ HMGA1,^[^ ^58^ ^]^ CRKL,^[^ ^59^ ^]^ EIF5A,^[^ ^60^ ^]^ and MCM7.^[^ ^61^ ^]^ Moreover, the Gene Ontology (GO) enrichment analysis was performed based on these differential proteins. As shown in Figure 5d, most of the pathways are associated with biological processes such as cytoplasmic translation, protein folding, and telomere lengthening regulation, indicating that the cytotoxicity of immune cells against tumor cells was directed toward the diminution of various fundamental vital activities of the tumor cells.

Similarly, the light channel data was also analyzed by UMAP clustering and differential analysis to investigate the proteome differences between the NK92MI cells with different cytotoxic activity. Two primary NK92MI cell clusters were identified using UMAP after normalization and batch correction. As shown in Figure 5e, 6 out of 10 NK92MI cells exhibiting killing activity were attributed to cluster L1 (n = 24), and 4 were attributed to cluster L2 (n = 19), indicating that cytotoxicity was not a significant factor in the differentiation of NK92MI cells into two clusters. To further analyze the differences between the two clusters, a total of 129 proteins with differential abundance were screened, among which 76 proteins were observed to be up‐regulated in cluster L1 relative to cluster L2, while 53 proteins were observed to be down‐regulated (Figure 5f). The differential proteins were enriched by GO enrichment analysis for the regulation of humoral immune responses, endopeptidase activity, proteolysis, and peptidase activity (Figure 5g). Differential expression was observed for proteins involved in NK cell‐mediated cytotoxicity pathways, as well as for proteins related to the formation and function of the immune synapse, including PARD3,^[^ ^62^ ^]^ LCP2,^[^ ^63^ ^]^ VCL,^[^ ^64^ ^]^ CASP3,^[^ ^65^ ^]^ APOA1, PON1, PTPN1, and COX2 (Figure 5h). It has been reported that increased production of reactive oxygen species (ROS) enables NK‐cell‐mediated cytolysis.^[^ ^66^ ^]^ However, excessive ROS may lead to cellular damage, which can be prevented by up‐regulating apolipoprotein A1 (APOA1) and paraoxonase‐1 (PON1).^[^ ^66^ ^]^ This helps ensure a normal immune response and prevents ROS damage to NK cells. In addition, the protein tyrosine phosphatase (PTPN1)^[^ ^67^ ^]^ and cyclooxygenase‐2 (COX‐2)^[^ ^68^ ^]^ are central regulators of inflammation, and their downregulation in immune cells promotes anti‐tumor immunity.

## 3 Discussion

In this work, we constructed an immune‐cancer interaction multi‐dimensional profiling platform mainly based on the microfluidic single cell‐pair approach and the respective proteomic analysis approach for single cell‐pairs based on stable‐isotope labeling. The workflow encompassed single‐cell sorting and capture, single‐cell pairing, cell–cell interaction monitoring, single cell‐pair retrieval and simultaneous sample pretreatment, LC‐MS/MS analysis, and respective data processing. It integrated cellular interaction fluorescence image information with in‐depth proteomics data to explore the heterogeneity of immune responses.

This platform utilized the novel microfluidic cell pairing approach based on the spatial trapping effect of the capillary probe tip to achieve accurate capture and pairing of single cells with high reliability and success rate. Compared to previous approaches relying on random distribution,^[^ ^69^, ^70^ ^]^ the integration of the capillary probe‐based microfluidic manipulation module with the microscopic fluorescence imaging module enabled WYSIWYG (What You See Is What You Get)‐style operation not only in the selection, capture and pairing of the target single cells but also in the monitoring and retrieval of the single cell‐pairs. The platform provided a solid technical guarantee for achieving multidimensional analysis of cell interactions. Compared to the commonly‐used microfluidic chip systems,^[^ ^23^ ^]^ the present platform could achieve the convenient retrieval of specific cell pairs at any point in time and perform the subsequent complex single‐cell proteomic analysis.

This work realized the combination of the cellular interaction behavior monitoring and deep proteomic analysis for immune‐cancer interaction profiling at the single‐cell level for the first time. Deep and quantitative proteome datasets can provide more direct biological information. Meanwhile, studies have shown that NK cells interact with tumor cells by recognizing and grasping tumor cells through the formation of the immune synapse, and then lysing and killing the target cells by the release of granzymes through the immune synapse.^[^ ^47^ ^]^ In the deep proteomic analysis of cell pairs, simply roughly using external forces to separate the interacting immune cells and tumor cells for direct single cell‐pair proteomic analysis is inappropriate since the destruction of the close cell–cell association would result in the cell damage and protein loss. Consequently, this work applied the respective proteomic analysis approach based on SILAC labeling, which enabled the analysis of differential protein expression corresponding to the phenotypes of different cells at the single‐cell level. This approach thus allowed for the in‐depth proteomic analysis of the cell pairs in a precise and comprehensive manner, which could be applied not only for other kinds of immune‐cancer cell interaction like T cells and their target cells, but also for different kinds of single cell‐pair interaction studies.

In summary, this platform offers a novel approach to studying cellular interactions at the single cell proteomic level. It provides an effective pairing and analysis tool with substantial advantages in terms of precision, reliability, utility, and compatibility. Although the current throughput of this platform for cell pairing was 3 min/cell pair, which was lower than those of the most existing methods, it still matched the throughput of tens of samples per day of most current proteomic analysis systems. Actually, the time for forming single‐cell pairs could be further reduced, e.g. reaching 60 s per cell pair, by increasing the moving speed of the translational stages and shortening the system optical focusing time. The overall analytical throughput of the system for proteomic analysis of single‐cell pairs was ca. 20 single‐cell pairs per day, with the major rate‐limiting step being the time taken for the LC‐MS/MS analysis. Future attempts to introduce high‐speed, high‐sensitive LC‐MS/MS systems could further improve the overall throughput of this platform. In addition to proteomic analysis, multi‐omics analysis techniques can be employed to obtain a more comprehensive understanding of the molecular basis underlying the heterogeneity of immune responses, such as transcriptomics and metabolomics. The present platform shows the substantial ability to flexibly manipulating cells and microfluids to accomplish cell pairing, monitoring, sampling, multi‐step pretreatment, and in‐depth single‐cell proteomic analysis, which also offers the possibility of simultaneously analyzing secreted proteomes and intracellular proteomes at the single‐cell level, which will significantly enhance the understanding of cell–cell interactions at high resolution. These studies will be crucial in identifying new biomarkers or drug targets for tumor immunotherapy, cell therapy, and drug development.

## 4 Experimental Section

### Cell Culture and Labeling

The NK92MI (iCell‐h331) and K562 (iCell‐h118) cell lines were obtained from iCell Bioscience Co. (Shanghai, China). NK92MI cells were cultured in NK92MI cell special medium (iCell, Catalog no. iCell‐h331‐001b). Non‐SILAC (“light”) K562 cells were cultured in regular IMDM (iCell, Catalog no. iCell 0008) supplemented with 10% fetal bovine serum (FBS; VivaCell, Catalog no. C3820‐0500), while the SILAC‐labeled (“heavy”) K562 cells were cultured in the RPMI 1640 medium (Thermo Scientific, Catalog no. 88365) containing 10% dialyzed FBS (VivaCell, Catalog no. C3820‐0100), in which normal lysine and arginine were replaced with ^13^C_6_^15^N_2_ L‐lysine‐2HCl (Thermo Scientific, Catalog no. 88209) and ^13^C_6_^15^N_4_ L‐arginine‐HCl (Thermo Scientific, Catalog no. 89990). All cell lines were maintained in a cell incubator at 37 °C with 5% CO_2_. The medium was replaced every 2–3 days. The K562 cells were kept in the heavy medium for 2 weeks, during which the labeling efficiencies of single K562 cells were measured. After culturing, the cells were harvested at 70% confluency for further treatment.

### Cell Staining

In order to distinguish NK92MI cells and K562 cells, 5 µM Calcein Red AM (Invitrogen, Catalog no. C34851) and 10 µM Cell Tracker Blue dyes (Invitrogen, Catalog no. C2110) were chosen to stain them, respectively. For staining, ca. 1 million cells were first washed with phosphate buffered saline (PBS), then resuspended in the freshly prepared dye solution in‐PBS (1 mL) and incubated at 37 °C for 30 min. Then, the cells were washed twice and resuspended in NK92MI cell special medium containing 2.5 µM SYTOX green (Invitrogen, Catalog no. S7020). The stained cells were then proceeded for single‐cell pairing.

### Single‐Cell Pairing

The single cell‐pair manipulation module was established based on the sequential operation droplet array (SODA) system previously developed by the authors’ group for single‐cell capture and droplet manipulation.^[^ ^45^, ^46^ ^]^ The module setup comprised an inverted microscope (ECLIPSE Ti2, Nikon Co., Tokyo, Japan) for observing and identifying cells, an automated *x‐y* translation stage for controlling the horizontal movement of the 384‐well plate and insert tubes, a capillary probe with a tapered tip (5.5 cm length, 375 µm o.d., 150 µm i.d., tip inner diameter, 35 µm i.d., Yongnian Ruifeng Co., Handan, China) connected to a home‐made syringe pump fixed on an automated *z* translation stage for nanoliter‐scale liquid handling, and a system control module. The capillary probes were fabricated by first using the laser pulling method to obtain a tapped probe tip, and then grinding the probe tip end with sandpapers. During the grinding process, the inner diameter of the probe tip was continuously monitored with a microscope to accurately measure the inner diameter of the tip end. Since we obtained the desired inner diameter of the probe tip by changing the grit types of the sandpapers and the grinding time under the monitoring of the microscope, the errors of the tip inner diameters of the probes could be controlled in the range of ±1 µm (Figure S9, Supporting Information).

For single‐cell pairing, 60 µL of NK92MI cell special medium containing viability dyes Sytox Green was loaded into a 384‐well plate (NEST Biotech, Catalog no.761001) in advance for further cell co‐culture. The 384‐well plates were chosen because they could facilitate the observation and positioning of the cell pairs, as well as avoid cross‐interference between different cell pairs. First, 5 µL of two different cell suspensions with a cell density of approximately 1 × 10^6^ cells/mL were added respectively into the different wells of the 384‐well plate. The target single NK92MI cell and single K562 cell were respectively selected after checking their viability by bright field and fluorescence observation. Then the tapered tip of the capillary probe was controlled to align the target cell by moving the *x‐y* and *z* translational stages and sucking the single NK92MI cell and K562 cell sequentially into the probe channel by aspirating 3 nL of each cell suspension using the syringe pump. Finally, 8 nL of cell suspension containing the two single cells was dispensed from the probe tip to the bottom of a plate well for single cell pair. The suspension of cells flowed out of the probe tip through the gap (ca. 5 µm) between the capillary tip and the bottom of the plate well, while the capillary tip wall trapped the two single cells, resulting in the acquisition of a single NK92MI‐K562 cell pair. During the single‐cell pairing process, the aspirating and dispensing flow rates were both set to 1 nL s^−1^.

### Fluorescence Image Acquisition and Analysis

After the single‐cell pairing, the 384‐well plate was placed in the live‐cell workstation (Tokai Hit, STXG) with 5% CO_2_ and a temperature of 37 °C. Time‐lapse images, including bright field and fluorescence images, were automatically acquired every 5 min for 4 h at multiple positions using a confocal microscope (A1R HD25, Nikon Co., Tokyo, Japan) with a 10× objective and filter wavelengths of 405, 488, and 561 nm. Usually, the first imaging was initiated 5 min after the cell pair was formed due to the need for microscope preparation and adjustment, and this time point was marked as t = 0 h. The time‐lapse image processing and data analysis were performed using the NIS‐Elements AR 5.01 software. The cytotoxicity of NK92MI cells was quantified as the percentage of the K562 cells with green fluorescence (stained with STYTOX Green) to the total number of the K562 cells.

### Sample Pretreatment Before Proteomic Analysis

Each cell pair was washed in PBS and then transferred into the insert tube. The blank samples were PBS suspension. We used the single‐cell sample pretreatment workflow developed by the authors’ group.^[^ ^44^ ^]^ Briefly, 400 nL of 0.3% (w/v) RapeGest (Waters Co., Milford, USA) was added into the insert tube containing the cell pair droplet and incubated at 60 °C for 20 min to lyse the cells. Then the mixture was cooled to room temperature, after which 400 nL of 10 mM Tris(2‐carboxyethyl) phosphine hydrochloride (TCEP) and 50 mM iodoacetamide (IAA) were added sequentially and incubated at room temperature for 20 min and 15 min for reduction and alkylation, respectively. Next, 400 nL of 50 µg mL^−1^ trypsin/lys‐C mixture was added and incubated at 37 °C for 2 h to digest the proteins. The reaction was terminated by the addition of 800 nL of 50% (v/v) formic acid (FA) solution.

### LC‐MS/MS Analysis

LC separations of the digested peptide samples were carried out using an EASY‐nLC 1200 LC system (Thermo Fisher Scientific Co., St. Louis, USA) at a mobile‐phase flow rate of 150 nL min^−1^ with a 21‐min gradient. The column‐packing procedure and liquid phase gradient settings followed the previously described method.^[^ ^44^ ^]^ Briefly, the analytical columns were packed with 1.7 µm C_18_ particles (120 Å pore size, Nanomicro Co., Suzhou, China) in‐lab using 50 µm i.d. fused silica capillaries (Agilent Co., Santa Clara, USA).

The separated peptides were detected by a trapped‐ion mobility time‐of‐flight mass spectrometer (timsTOF Pro, Bruker Co., Billerica, USA). The acquisition mode of diaPASEF was used in the analysis of single‐cell pair samples, with the mass scan range from m/z 375 to 1575, the isolation width of 25 m/z, the mobility 1/k0 range of 0.58–1.43, the collision‐induced dissociation (CID) collision energy of 20–47.3 eV and the cycle time of 1.68 s. All data were acquired under the positive mode and the spray voltage of ion source was set at 1.75 kV.

### MS Data Analysis

The raw data of proteomics were analyzed on Spectronaut software (Version 18.2.230802.50606) in library‐free search mode and searched against the UniProt Proteomes *Homo sapiens* database (accession: UP000005640, taxon ID: 9606, 20,422 entries, access date 2023‐03). False‐discovery rates (FDR) were controlled at 1% on both precursor and protein levels in the default settings. For SILAC samples, a two‐channel workflow was applied with non‐label in Channel 1 (Light) and Arg10 and Lys8 as labels in Channel 2 (Heavy). Uncheck “Cross Run Normalization” of the quantitation level. All other parameters were set to default.

### Statistical Analysis

The data in this paper were expressed as mean ± SD. All bioinformatics analysis and visualization were conducted by R (version 4.2.3). Proteins quantified in less than 70% cells were excluded and missing values were imputed with zeros. To eliminate interference introduced by the reagents, environment, and quantitative method, the protein quantity of the heavy channel was adjusted by subtracting the mean protein quantity of the heavy channel of the non‐labeled samples, while the value of the light channel was adjusted by subtracting the mean protein quantity of the light channel of the blank samples. The batch effects of samples were corrected by the ComBat function of the sva package (version 3.46.0).^[^ ^71^ ^]^ The Seurat package (version 4.3.0) was used for the uniform manifold approximation and projection (UMAP) analysis and unsupervised cluster analysis.^[^ ^72^ ^]^ Differential proteins were determined by Limma package (version 3.54.2),^[^ ^73^ ^]^ and the Benjamini‐Hochberg's method was used for adjustment of p values (p.adj <0.05), the value of which was used for both differential analysis and gene ontology (GO) enrichment analysis. GO enrichment was carried out using the clusterProfiler package (version 4.6.2).^[^ ^74^ ^]^

## Conflict of Interest

The authors declare no conflict of interest.

## Author Contributions

Q.Q.X., Y.R.J. contributed equally to this work. Q.F., Q.Q.X., and Y.R.J. conceived the idea. Q.Q.X., Y.R.J., J.B.C. and H.F.W. built the instrument. H.F.W. wrote the software. Q.Q.X., Y.X.C., and Q.X.F. cultured the cells. Q.Q.X. planned and performed experiments. Q.Q.X. and Y.R.J. provided nano‐LC columns. Q.Q.X., Y.R.J., Y.Y., and Q.F. contributed to data analysis and interpretation, as well as figure preparation. Q.F., Q.Q.X., Y.R.J., and Y.Y. wrote and revised the manuscript. Q.Q.X., Y.R.J., Y.Y., J.W., Y.X.C., Q.X.F., and Q.F. contributed to the supplementary experiments. Q.F., J.Z.P., and Y.Y. supervised the studies and acquired funding.

## Supporting information



Supporting Information

Supplemental Table 1

Supplemental Video 3

Supplemental Video 4

Supplemental Video 5

## Data Availability

The data that support the findings of this study are available in the supplementary material of this article.

## References

[1] O. K. Dagher , R. D. Schwab , S. K. Brookens , A. D. Posey Jr. , Cell 2023, 186, 1814. 37059073 10.1016/j.cell.2023.02.039

[2] P. S. Hegde , D. S. Chen , Immunity 2020, 52, 17. 31940268 10.1016/j.immuni.2019.12.011

[3] R. Satija , A. K. Shalek , Trends Immunol. 2014, 35, 219. 24746883 10.1016/j.it.2014.03.004 PMC4035247

[4] D. A. Lawson , K. Kessenbrock , R. T. Davis , N. Pervolarakis , Z. Werb , Nat. Cell Biol. 2018, 20, 1349. 30482943 10.1038/s41556-018-0236-7 PMC6477686

[5] T. Konry , S. Sarkar , P. Sabhachandani , N. Cohen , Annu. Rev. Biomed. Eng. 2016, 18, 259. 26928209 10.1146/annurev-bioeng-090215-112735

[6] L. Huang , Y. Chen , J. Zhou , Cell Rep. Phys. Sci. 2022, 3, 101129.

[7] N. Subedi , L. C. Van Eyndhoven , A. M. Hokke , L. Houben , M. C. Van Turnhout , C. V. C. Bouten , K. Eyer , J. Tel , Sci. Rep. 2021, 11, 17084. 34429486 10.1038/s41598-021-96609-9 PMC8385055

[8] J. L. Madrigal , N. G. Schoepp , L. Xu , C. S. Powell , C. L. Delley , C. A. Siltanen , J. Danao , M. Srinivasan , R. H. Cole , A. R. Abate , Proc. Natl Acad. Sci. USA 2022, 119, e2110867119. 35074872 10.1073/pnas.2110867119 PMC8812558

[9] S. E. Kim , H. Kim , J. Doh , Lab Chip. 2019, 19, 2009. 31065640 10.1039/c9lc00133f

[10] H. Tu , Z. Wu , Y. Xia , H. Chen , H. Hu , Z. Ding , F. Zhou , S. Guo , Analyst 2020, 145, 4138. 32409799 10.1039/d0an00110d

[11] K. Zhang , C. K. Chou , X. Xia , M. C. Hung , L. Qin , Proc. Natl Acad. Sci. USA 2014, 111, 2948. 24516129 10.1073/pnas.1313661111 PMC3939871

[12] L. Li , H. Wang , L. Huang , S. A. Michael , W. Huang , H. Wu , Anal. Chem. 2019, 91, 15908. 31741379 10.1021/acs.analchem.9b04370

[13] F. A. Shaik , C. Lewuillon , A. Guillemette , B. Ahmadian , C. Brinster , B. Quesnel , D. Collard , Y. Touil , L. Lemonnier , M. C. Tarhan , Lab Chip. 2022, 22, 908. 35098952 10.1039/d1lc01156a

[14] C. Wu , R. Chen , Y. Liu , Z. Yu , Y. Jiang , X. Cheng , Lab Chip. 2017, 17, 4008. 29115319 10.1039/c7lc01082f

[15] M. T. Chung , D. Nunez , D. Cai , K. Kurabayashi , Lab Chip. 2017, 17, 3664. 28967663 10.1039/c7lc00745k

[16] J. Liu , X. Lyu , Z. Zhou , L. Yang , J. Zeng , Y. Yang , Z. Zhao , R. Chen , X. Tong , J. Li , H. Liu , Y. Zou , ACS Appl. Mater. Interfaces 2023, 15, 17324. 36962257 10.1021/acsami.2c23003

[17] X. Wang , S. Chen , M. Kong , Z. Wang , K. D. Costa , R. A. Li , D. Sun , Lab Chip. 2011, 11, 3656. 21918752 10.1039/c1lc20653b

[18] T. Fang , W. Shang , C. Liu , J. Xu , D. Zhao , Y. Liu , A. Ye , Anal. Chem. 2019, 91, 9932. 31251569 10.1021/acs.analchem.9b01604

[19] F. Guo , P. Li , J. B. French , Z. Mao , H. Zhao , S. Li , N. Nama , J. R. Fick , S. J. Benkovic , T. J. Huang , Proc. Natl Acad. Sci. USA 2015, 112, 43. 25535339 10.1073/pnas.1422068112 PMC4291613

[20] T. Kosaka , S. Yamaguchi , S. Izuta , S. Yamahira , Y. Shibasaki , H. Tateno , A. Okamoto , J. Am. Chem. Soc. 2022, 144, 17980. 36126284 10.1021/jacs.2c07321

[21] D. J. Collins , A. Neild , A. deMello , A. Q. Liu , Y. Ai , Lab Chip. 2015, 15, 3439. 26226550 10.1039/c5lc00614g

[22] Y. Zhou , N. Shao , R. Bessa de Castro , P. Zhang , Y. Ma , X. Liu , F. Huang , R. F. Wang , L. Qin , Cell Rep. 2020, 31, 107574. 32348757 10.1016/j.celrep.2020.107574 PMC7583657

[23] L. Pang , J. Ding , X.‐X. Liu , H. Yuan , Y. Ge , J. Fan , S.‐K. Fan , Trends Anal. Chem. 2020, 129, 115940.

[24] A. M. Skelley , O. Kirak , H. Suh , R. Jaenisch , J. Voldman , Nat. Methods 2009, 6, 147. 19122668 10.1038/nmeth.1290 PMC3251011

[25] B. Dura , S. K. Dougan , M. Barisa , M. M. Hoehl , C. T. Lo , H. L. Ploegh , J. Voldman , Nat. Commun. 2015, 6, 5940. 25585172 10.1038/ncomms6940

[26] B. Dura , M. M. Servos , R. M. Barry , H. L. Ploegh , S. K. Dougan , J. Voldman , Proc. Natl Acad. Sci. USA 2016, 113, E3599. 27303033 10.1073/pnas.1515364113 PMC4932925

[27] T. J. Bechtel , T. Reyes‐Robles , O. O. Fadeyi , R. C. Oslund , Nat. Chem. Biol. 2021, 17, 641. 34035514 10.1038/s41589-021-00790-x

[28] B. A. Yang , T. M. Westerhof , K. Sabin , S. D. Merajver , C. A. Aguilar , Adv. Sci. 2021, 8, 2002825. 10.1002/advs.202002825 PMC7856891 33552865

[29] S. Im , D. Jang , G. Saravanakumar , J. Lee , Y. Kang , Y. M. Lee , J. Lee , J. Doh , Z. Y. Yang , M. H. Jang , W. J. Kim , Adv. Mater. 2020, 32, 2000020. 10.1002/adma.202000020 32319126

[30] Y. J. Yamanaka , C. T. Berger , M. Sips , P. C. Cheney , G. Alter , J. C. Love , Integr. Biol. 2012, 4, 1175. 10.1039/c2ib20167d 22945136

[31] P. E. Olofsson , E. Forslund , B. Vanherberghen , K. Chechet , O. Mickelin , A. R. Ahlin , T. Everhorn , B. Onfelt , Front. Immunol. 2014, 5, 80. 24639676 10.3389/fimmu.2014.00080 PMC3945532

[32] X. Tang , X. Liu , P. Li , F. Liu , M. Kojima , Q. Huang , T. Arai , Anal. Chem. 2020, 92, 11607. 32605365 10.1021/acs.analchem.0c01148

[33] E. Papalexi , R. Satija , Nat. Rev. Immunol. 2018, 18, 35. 28787399 10.1038/nri.2017.76

[34] J. C. Rieckmann , R. Geiger , D. Hornburg , T. Wolf , K. Kveler , D. Jarrossay , F. Sallusto , S. S. Shen‐Orr , A. Lanzavecchia , M. Mann , F. Meissner , Nat. Immunol. 2017, 18, 583. 28263321 10.1038/ni.3693

[35] Z. Shen , H. Zhao , H. Yao , X. Pan , J. Yang , S. Zhang , G. Han , X. Zhang , Chem. Sci. 2022, 13, 1641. 35282636 10.1039/d1sc06366a PMC8827047

[36] L. Li , S. Yan , B. Lin , Q. Shi , Y. Lu , Adv. Cancer Res. 2018, 139, 185. 29941105 10.1016/bs.acr.2018.04.006

[37] Y. Lu , J. J. Chen , L. Mu , Q. Xue , Y. Wu , P. H. Wu , J. Li , A. O. Vortmeyer , K. Miller‐Jensen , D. Wirtz , R. Fan , Anal. Chem. 2013, 85, 2548. 23339603 10.1021/ac400082e PMC3589817

[38] M. Elitas , K. Brower , Y. Lu , J. J. Chen , R. Fan , Lab Chip. 2014, 14, 3582. 25057779 10.1039/c4lc00676c PMC4145007

[39] J. Deng , Y. Ji , F. Zhu , L. Liu , L. Li , X. Bai , H. Li , X. Liu , Y. Luo , B. Lin , Y. Lu , Proc. Natl Acad. Sci. USA 2022, 119, e2200944119. 36288285 10.1073/pnas.2200944119 PMC9636946

[40] L. Li , H. Su , Y. Ji , F. Zhu , J. Deng , X. Bai , H. Li , X. Liu , Y. Luo , B. Lin , T. Liu , Y. Lu , Adv. Sci. 2023, 10, e2301018. 10.1002/advs.202301018 PMC10323649 37186381

[41] Z. Y. Li , M. Huang , X. K. Wang , Y. Zhu , J. S. Li , C. C. L. Wong , Q. Fang , Anal. Chem. 2018, 90, 5430. 29551058 10.1021/acs.analchem.8b00661

[42] H. Specht , E. Emmott , A. A. Petelski , R. G. Huffman , D. H. Perlman , M. Serra , P. Kharchenko , A. Koller , N. Slavov , Genome. Biol. 2021, 22, 50. 33504367 10.1186/s13059-021-02267-5 PMC7839219

[43] A. D. Brunner , M. Thielert , C. Vasilopoulou , C. Ammar , F. Coscia , A. Mund , O. B. Hoerning , N. Bache , A. Apalategui , M. Lubeck , S. Richter , D. S. Fischer , O. Raether , M. A. Park , F. Meier , F. J. Theis , M. Mann , Mol. Syst. Biol. 2022, 18, e10798. 35226415 10.15252/msb.202110798 PMC8884154

[44] Y. Wang , Z. Y. Guan , S. W. Shi , Y. R. Jiang , J. Zhang , Y. Yang , Q. Wu , J. Wu , J. B. Chen , W. X. Ying , Q. Q. Xu , Q. X. Fan , H. F. Wang , L. Zhou , L. Wang , J. Fang , J. Z. Pan , Q. Fang , Nat. Commun. 2024, 15, 1279. 38341466 10.1038/s41467-024-45659-4 PMC10858870

[45] Y. Zhu , Y. X. Zhang , L. F. Cai , Q. Fang , Anal. Chem. 2013, 85, 6723. 23763273 10.1021/ac4006414

[46] Z. Dong , Q. Fang , TrAC, Trends Anal. Chem. 2020, 124, 115812.

[47] J. S. Orange , Nat. Rev. Immunol. 2008, 8, 713. 19172692 10.1038/nri2381 PMC2772177

[48] P. T. Hamilton , B. R. Anholt , B. H. Nelson , Nat. Rev. Immunol. 2022, 22, 765. 35513493 10.1038/s41577-022-00719-y

[49] X. Zheng , Z. Hou , Y. Qian , Y. Zhang , Q. Cui , X. Wang , Y. Shen , Z. Liu , Y. Zhou , B. Fu , R. Sun , Z. Tian , G. Huang , H. Wei , Nat. Immunol. 2023, 24, 802. 36959292 10.1038/s41590-023-01462-9

[50] S. E. Ong , M. Mann , Nat. Protoc. 2006, 1, 2650. 17406521 10.1038/nprot.2006.427

[51] L. Wexler , D. H. Bergel , I. T. Gabe , G. S. Makin , C. J. Mills , Circ. Res. 1968, 23, 349. 5676450 10.1161/01.res.23.3.349

[52] B. Vanherberghen , P. E. Olofsson , E. Forslund , M. Sternberg‐Simon , M. A. Khorshidi , S. Pacouret , K. Guldevall , M. Enqvist , K. J. Malmberg , R. Mehr , B. Onfelt , Blood 2013, 121, 1326. 23287857 10.1182/blood-2012-06-439851

[53] K. Guldevall , L. Brandt , E. Forslund , K. Olofsson , T. W. Frisk , P. E. Olofsson , K. Gustafsson , O. Manneberg , B. Vanherberghen , H. Brismar , K. Karre , M. Uhlin , B. Onfelt , Front. Immunol. 2016, 7, 119. 27092139 10.3389/fimmu.2016.00119 PMC4820656

[54] S. Antona , I. Platzman , J. P. Spatz , ACS Omega 2020, 5, 24674. 33015484 10.1021/acsomega.0c03264 PMC7528335

[55] M. Vogt , N. Dienstbier , J. Schliehe‐Diecks , K. Scharov , J. W. Tu , P. Gebing , J. Hogenkamp , B. S. Bilen , S. Furlan , D. Picard , M. Remke , L. Yasin , D. Bickel , M. Kalia , A. Iacoangeli , T. Lenz , K. Stuhler , A. A. Pandyra , J. Hauer , U. Fischer , R. Wagener , A. Borkhardt , S. Bhatia , Cell Death Dis. 2023, 14, 799. 38057328 10.1038/s41419-023-06337-3 PMC10700369

[56] Y. Peng , Z. Huang , F. Zhou , T. Wang , K. Mou , W. Feng , Cell Commun. Signal. 2021, 19, 71. 34217296 10.1186/s12964-021-00752-9 PMC8254927

[57] D. Pan , W. Yang , Y. Zeng , W. Li , K. Wang , L. Zhao , J. Li , Y. Ye , Q. Guo , Cell Signal 2021, 84, 110038. 33984486 10.1016/j.cellsig.2021.110038

[58] M. De Martino , F. Esposito , A. Fusco , Hematol. Oncol. 2022, 40, 2. 34637548 10.1002/hon.2934 PMC9293314

[59] J. Yu , W. X. Chen , W. J. Xie , R. W. Chen , D. Q. Lin , W. W. You , W. L. Ye , H. Q. Zhang , D. H. Lin , J. P. Xu , J. Clin. Lab. Anal. 2021, 35, e23817. 34114685 10.1002/jcla.23817 PMC8373353

[60] S. Balabanov , A. Gontarewicz , P. Ziegler , U. Hartmann , W. Kammer , M. Copland , U. Brassat , M. Priemer , I. Hauber , T. Wilhelm , G. Schwarz , L. Kanz , C. Bokemeyer , J. Hauber , T. L. Holyoake , A. Nordheim , T. H. Brummendorf , Blood 2007, 109, 1701. 17008552 10.1182/blood-2005-03-037648

[61] L. Tian , B. Chen , J. Liu , J. Cheng , G. Xia , Blood 2015, 126, 5554.

[62] M. Yassin , S. M. Russell , Curr. Opin. Immunol. 2016, 39, 143. 26945468 10.1016/j.coi.2016.02.004

[63] S. Hidano , H. Sasanuma , K. Ohshima , K. Seino , L. Kumar , K. Hayashi , M. Hikida , T. Kurosaki , M. Taniguchi , R. S. Geha , D. Kitamura , R. Goitsuka , Int. Immunol. 2008, 20, 345. 18203684 10.1093/intimm/dxm150

[64] E. M. Mace , J. Zhang , K. A. Siminovitch , F. Takei , Blood 2010, 116, 1272. 20472831 10.1182/blood-2009-12-261487

[65] Y. P. Milyutina , V. A. Mikhailova , K. M. Pyatygina , E. S. Demidova , D. A. Malygina , T. E. Tertychnaia , A. V. Arutjunyan , D. I. Sokolov , S. A. Selkov , Biochemistry 2019, 84, 1186. 31694514 10.1134/S0006297919100079

[66] G. Morris , M. Gevezova , V. Sarafian , M. Maes , Cell Mol. Immunol. 2022, 19, 1079. 36056148 10.1038/s41423-022-00902-0 PMC9508259

[67] C. K. Baumgartner , H. Ebrahimi‐Nik , A. Iracheta‐Vellve , K. M. Hamel , K. E. Olander , T. G. R. Davis , K. A. McGuire , G. T. Halvorsen , O. I. Avila , C. H. Patel , S. Y. Kim , A. V. Kammula , A. J. Muscato , K. Halliwill , P. Geda , K. L. Klinge , Z. Xiong , R. Duggan , L. Mu , M. D. Yeary , J. C. Patti , T. M. Balon , R. Mathew , C. Backus , D. E. Kennedy , A. Chen , K. Longenecker , J. T. Klahn , C. L. Hrusch , N. Krishnan , et al., Nature 2023, 622, 850. 37794185 10.1038/s41586-023-06575-7 PMC10599993

[68] B. Liu , L. Qu , S. Yan , Cancer Cell Int. 2015, 15, 106. 26549987 10.1186/s12935-015-0260-7 PMC4635545

[69] H. N. Joensson , H. A Svahn , Angew. Chem., Int. Ed. 2012, 51, 12176. 10.1002/anie.201200460 23180509

[70] S.‐H. Kim , G. H. Lee , J. Y. Park , Biomed. Eng. Lett. 2013, 3, 131.

[71] J. T. Leek , W. E. Johnson , H. S. Parker , A. E. Jaffe , J. D. Storey , Bioinformatics 2012, 28, 882. 22257669 10.1093/bioinformatics/bts034 PMC3307112

[72] Y. Hao , S. Hao , E. Andersen‐Nissen , W. M. 3rd Mauck , S. Zheng , A. Butler , M. J. Lee , A. J. Wilk , C. Darby , M. Zager , P. Hoffman , M. Stoeckius , E. Papalexi , E. P. Mimitou , J. Jain , A. Srivastava , T. Stuart , L. M. Fleming , B. Yeung , A. J. Rogers , J. M. McElrath , C. A. Blish , R. Gottardo , P. Smibert , R. Satija , Cell 2021, 184, 3573. 34062119 10.1016/j.cell.2021.04.048 PMC8238499

[73] G. K. Smyth , Stat. Appl. Genet. Mol. Biol. 2004, 3, 1. 10.2202/1544-6115.1027 16646809

[74] T. Wu , E. Hu , S. Xu , M. Chen , P. Guo , Z. Dai , T. Feng , L. Zhou , W. Tang , L. Zhan , X. Fu , S. Liu , X. Bo , G. Yu , Innovation 2021, 2, 100141. 34557778 10.1016/j.xinn.2021.100141 PMC8454663

