# Supplementary material for: Single Cell‐Pair Proteomics for Decoding Immune‐Cancer Cell Interactions
Source: Adv Sci (Weinh). 2025 Jan 22;12(11):2414769. doi: 10.1002/advs.202414769 (PMC11923901; doi:10.1002/advs.202414769)
Supplement: Supplementary file 1 — Supporting Information [file ADVS-12-2414769-s001.pdf]

ADVANCED SCIENCE
Open Access

# Supporting Information

for *Adv. Sci.*, DOI 10.1002/advs.202414769

Single Cell-Pair Proteomics for Decoding Immune-Cancer Cell Interactions

*Qin-Qin Xu, Yi-Rong Jiang, Jian-Bo Chen, Jie Wu, Yi-Xue Chen, Qian-Xi Fan, Hui-Feng Wang, Yi Yang*, Jian-Zhang Pan* and Qun Fang**

# Supplementary Information

## Single cell-pair proteomics for decoding immune-cancer cell interactions

Qin-Qin Xu[1#], Yi-Rong Jiang[1#], Jian-Bo Chen[1,2], Jie Wu[1], Yi-Xue Chen[1], Qian-Xi Fan[1], Hui-Feng Wang[1,3], Yi Yang[2,4✉], Jian-Zhang Pan[1,2,4✉], and Qun Fang[1,2,3,4,5✉]

[1] Institute of Microanalytical Systems, Department of Chemistry, Zhejiang University, Hangzhou, 310058, China

[2] Single-cell Proteomics Research Center, ZJU-Hangzhou Global Scientific and Technological Innovation Center, Hangzhou, 311200, China

[3] Key Laboratory of Excited-State Materials of Zhejiang Province, Zhejiang University, Hangzhou, 310007, China

[4] Engineering Research Center of Functional Materials Intelligent Manufacturing of Zhejiang Province, Hangzhou, 311200, China

[5] Key Laboratory for Biomedical Engineering of Ministry of Education, Cancer Center, Zhejiang University, Hangzhou, 310007, China

[#] These authors contributed equally: Qin-Qin Xu, Yi-Rong Jiang.

✉e−mail: fangqun@zju.edu.cn; kelvonpan@zju.edu.cn; y_yi@zju.edu.cn

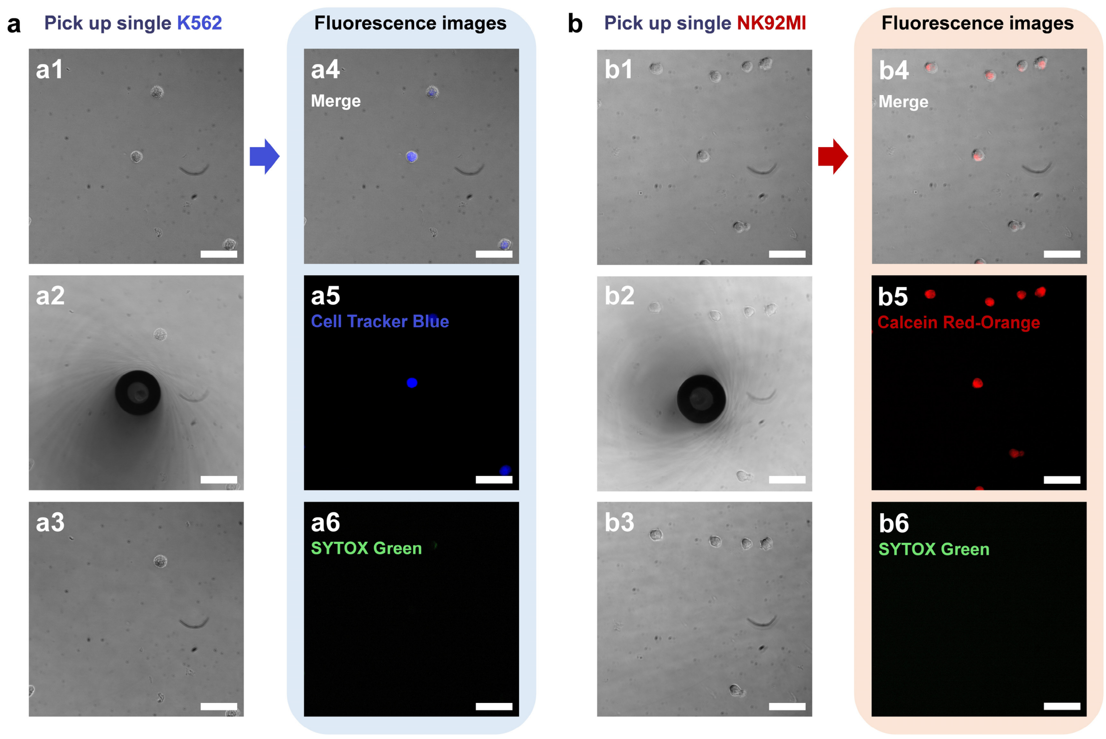


**Figure S1. Typical bright-field and fluorescence images of the precise single-cell capture process for a single K562 cell (a) and a single NK92MI cell (b).** (a1) A single target K562 cell was selected; (a2) The probe tip was aligned to the target K562 cell; (a3) After the target K562 cell was aspirated into the probe channel, it could not be observed in the field of view; (a4) The merged fluorescence image of the single target K562 cell; (a5, a6) The presence of blue fluorescence (a5) and the absence of green fluorescence (a6) indicated that the single target K562 cell exhibited good viability. (b1) A single target NK92MI cell was selected; (b2) The probe tip was aligned to the target NK92MI cell; (b3) After the target NK92MI cell was aspirated into the probe channel, it could not be observed in the field of view. (b4) The merged fluorescence image of the single target NK92MI cell; (b5, b6) The presence of red fluorescence (b5) and the absence of green fluorescence (b6) indicated that the single target NK92MI cell exhibited good viability.

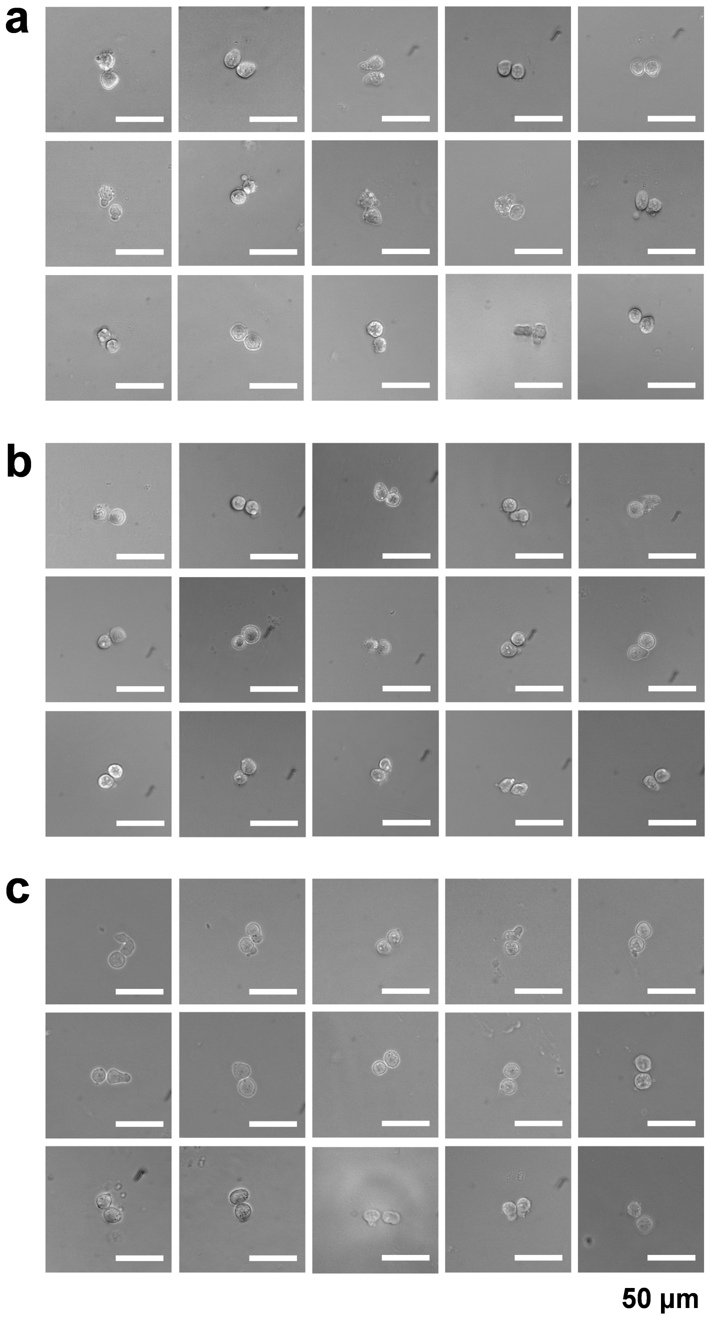


**Figure S2. Series of bright-field images of 45 cell pairs formed using three capillary probes (a, b, c) with a probe tip inner diameter of 35 µm and the single cell-pair manipulation module.** 15 consecutive single-cell pairings were performed for each capillary probe at a flow rate of 1 nL/s, achieving a high pairing success rate of 93% **(a)**, 100% **(b)**, and 100% **(c)**. The only unsuccessful case is as shown in the image in the second row and first column of **(a)**, where the two cells were not in close contact.

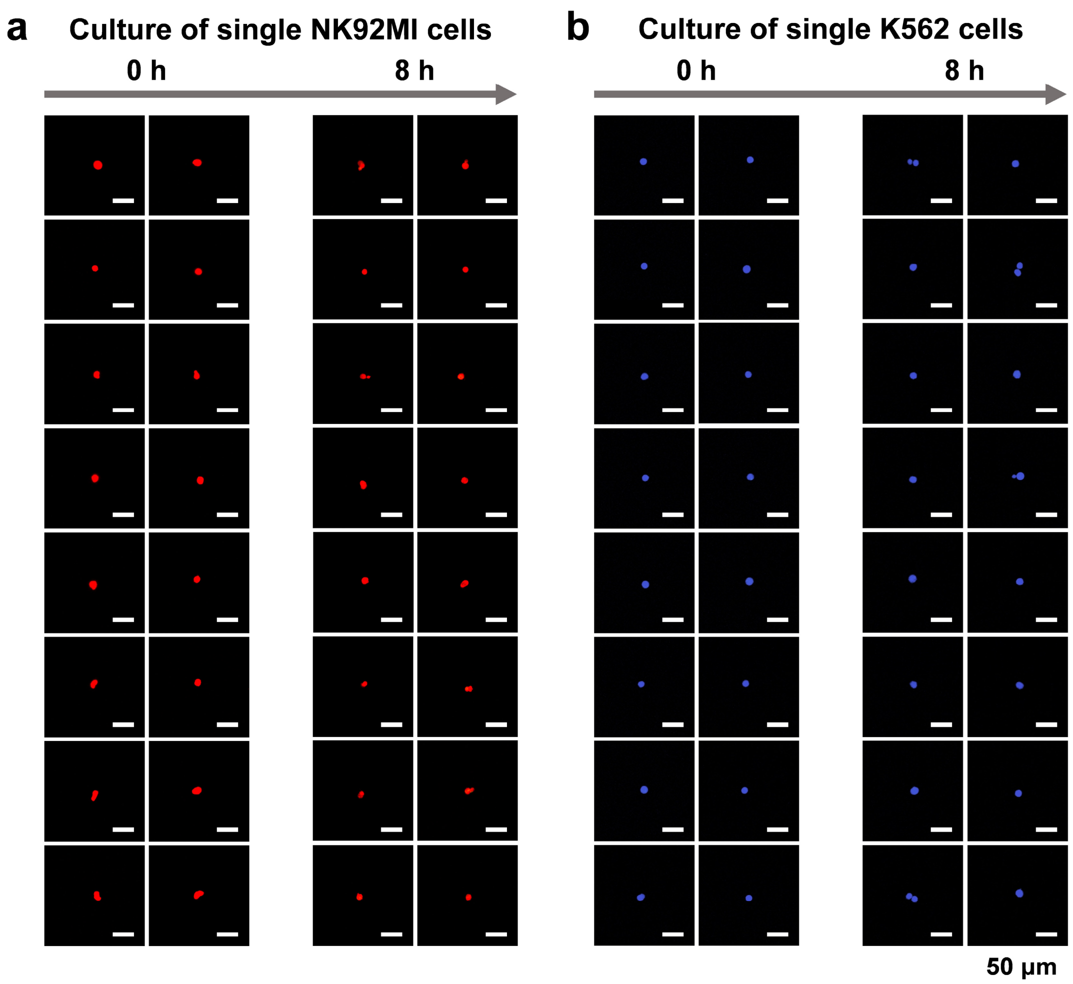


**Figure S3. Evaluation of the impact of the single cell-pair operation on the cell viability. (a)** Fluorescence images of single NK92MI cells in culture at 0 and 8 h after the single cell capture and dispensing operation; **(b)** Fluorescence images of single K562 cells in culture at 0 and 8 h after the single cell capture and dispensing operation. The single cells were picked up at a flow rate of 1 nL/s and dispensed into 60 μL NK92MI cell special medium containing Sytox Green and continuously monitored in the live-cell workstation for 8 h. These images showed that the single NK92MI cells and single K562 cells could retain their viability and even proliferation was observed in some single cells.

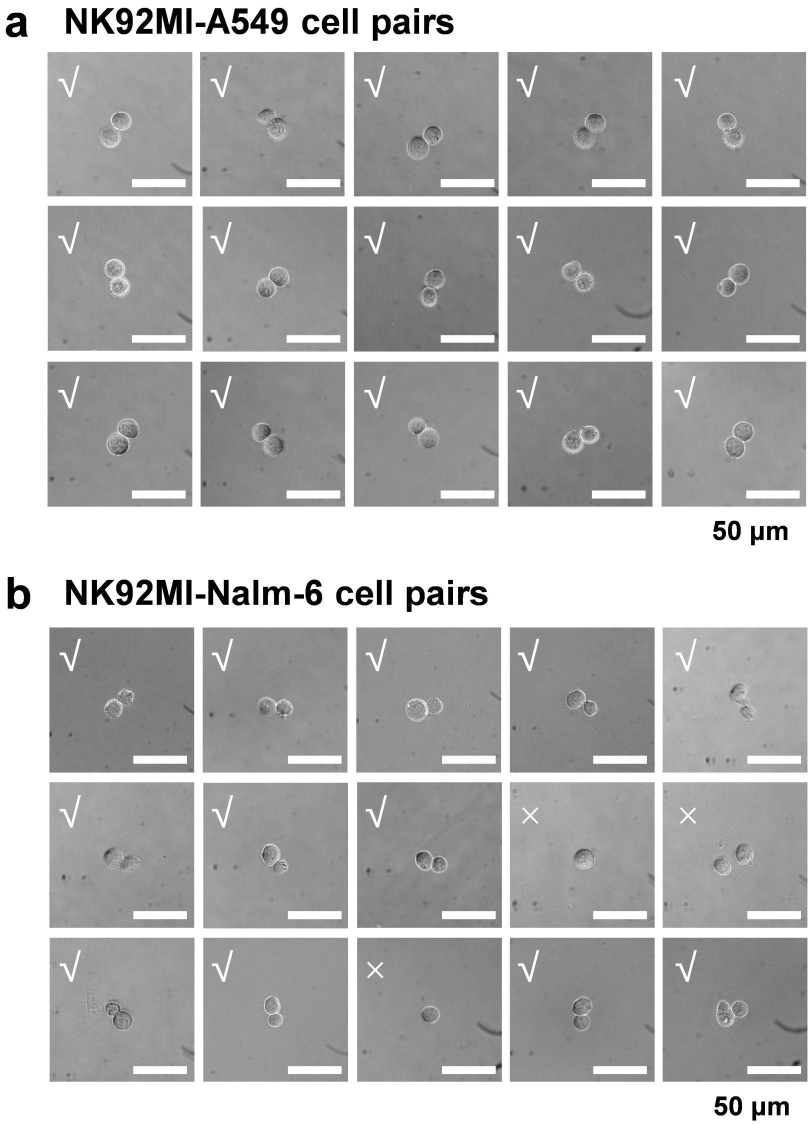


**Figure S4. Series of bright-field images of NK92MI-A549 cell pairs and NK92MI-Nalm-6 cell pairs formed under the optimized conditions. (a)** 15 consecutive single-cell pairings for NK92MI and A549 cells were performed using a capillary probe with the probe tip inner diameter of 37 μm at a flow rate of 1 nL/s, achieving a high pairing success rate of 100% (15/15). **(b)** 15 consecutive single-cell pairings NK92MI and Nalm-6 cells were performed using a capillary probe with the probe tip inner diameter of 33 μm at a flow rate of 1 nL/s, achieving a high pairing success rate of 80% (12/15).

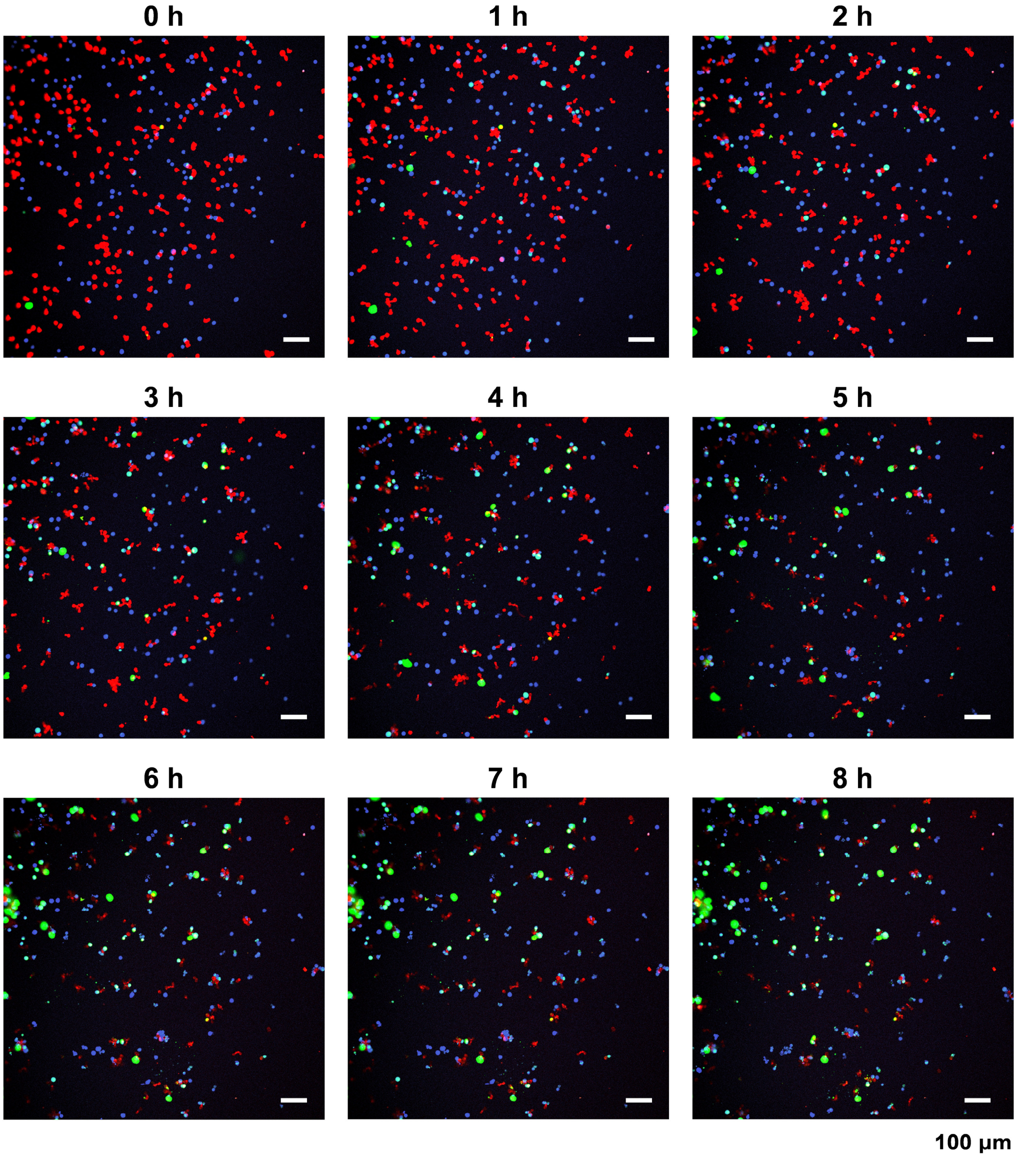


**Figure S5. A series of typical fluorescence images captured during the monitoring of the immune-cancer interactions in a bulk mixed sample of NK92MI and K562 cells.** The NK92MI cells and K562 cells were co-cultured at a ratio of 1:1. A total of 8 h of incubation was performed. The number of cell-killing events decreased over time during the 8-h interaction.

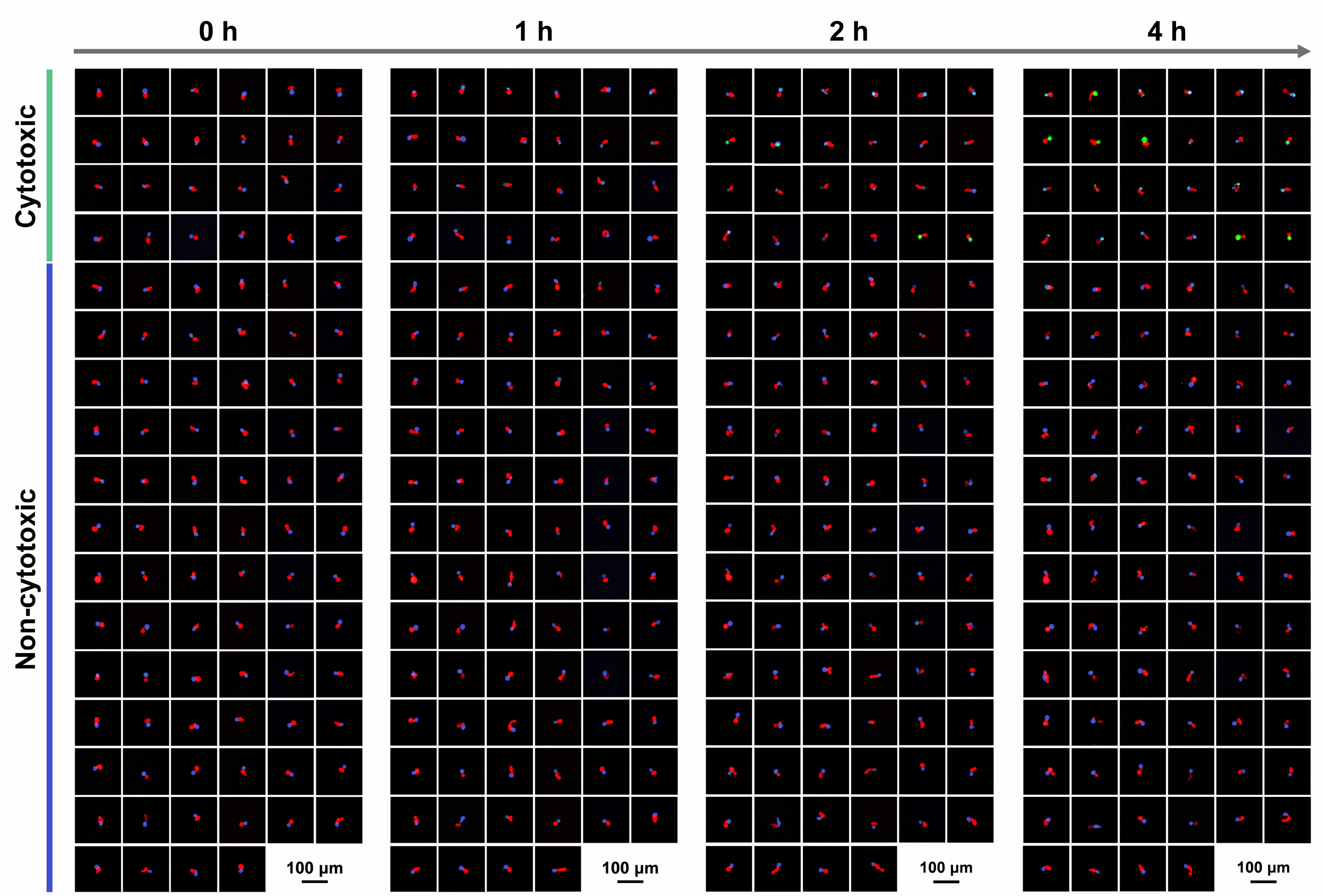


**Figure S6. Confocal images of 24 NK92MI-K562 cell pairs showing cytotoxic results and 76 NK92MI-K562 cell pairs showing non-cytotoxic results during the 4 h co-culture and interaction process.** The fluorescence images of each NK92MI-K562 cell pair were captured at 0, 1, 2, and 4-h time points. For those cell pairs showing cytotoxic results, the fluorescence of the K562 cells in the cell pairs transitioned from blue to green during the interaction, indicating apoptosis due to the NK cells' killing effect.

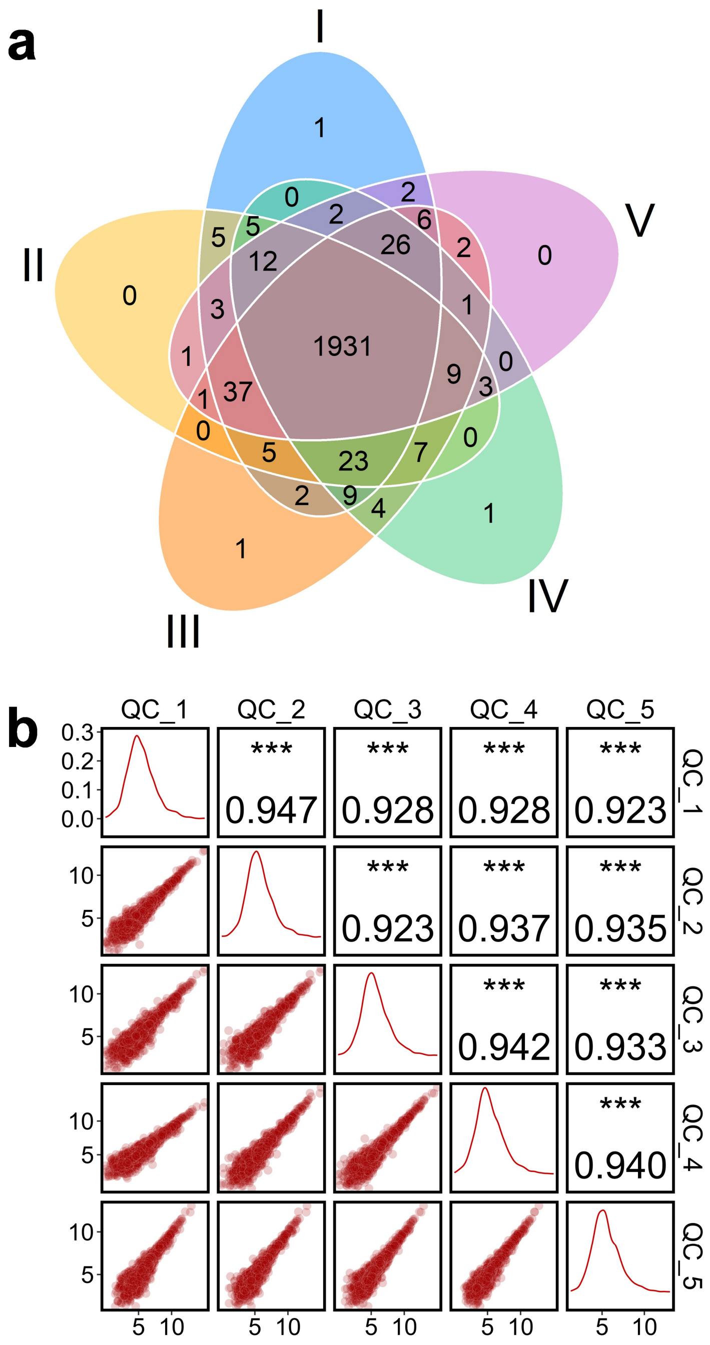


**Figure S7. The quantitative repeatability of the LC-MS/MS method. (a)** Venn diagram of the quantified protein groups in the QC sample (n = 5) under the DIA mode. There were 92% of the total protein groups quantified in all runs. **(b)** Correlation analysis of proteome expressions in the QC sample (n = 5) under the DIA mode. The correlation coefficients (CCs) of proteome expressions in the QC sample were all higher than 0.92.

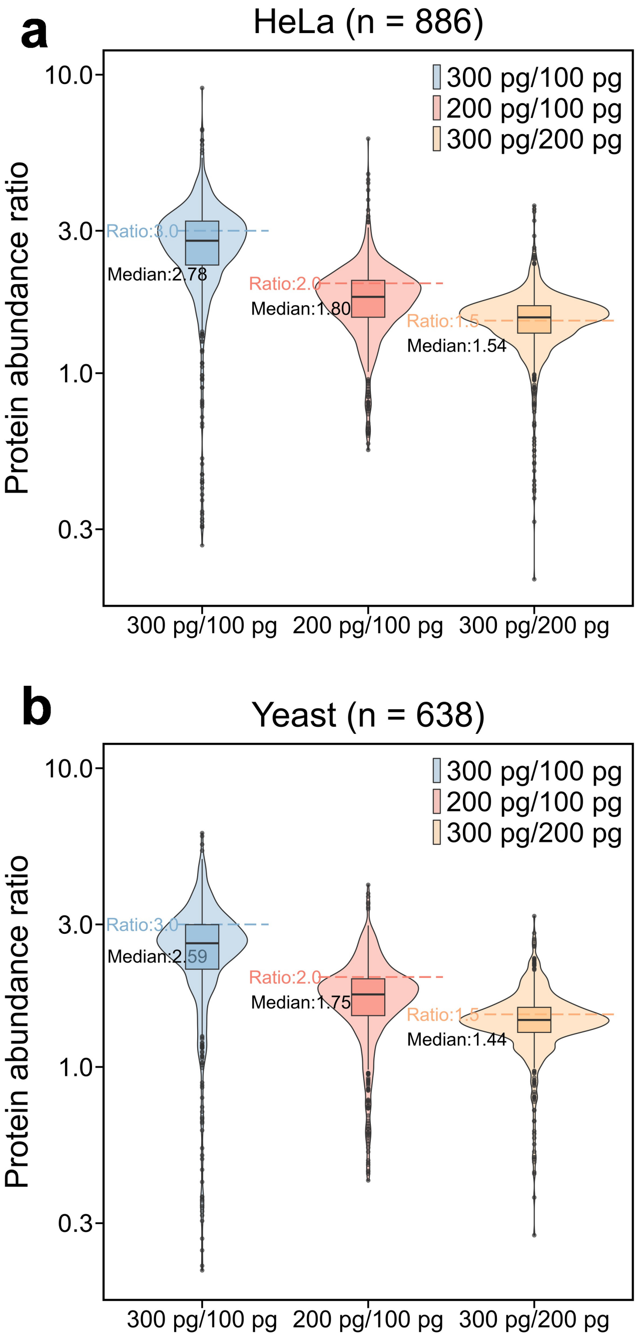


**Figure S8. The quantitative accuracy of the LC-MS/MS method. (a, b)** The benchmark experiment used the mixtures of digested HeLa and yeast samples in different proportions of 3:1, 2:2, and 1:3 (n = 3) with a total amount of proteins of 400 pg. The relative difference values between the medians of the protein abundance ratios and the theoretical calculated values were from 2.5% to 9.9% for the HeLa samples **(a)** and from 4.2% to 13.5% for the yeast samples **(b)**, respectively.

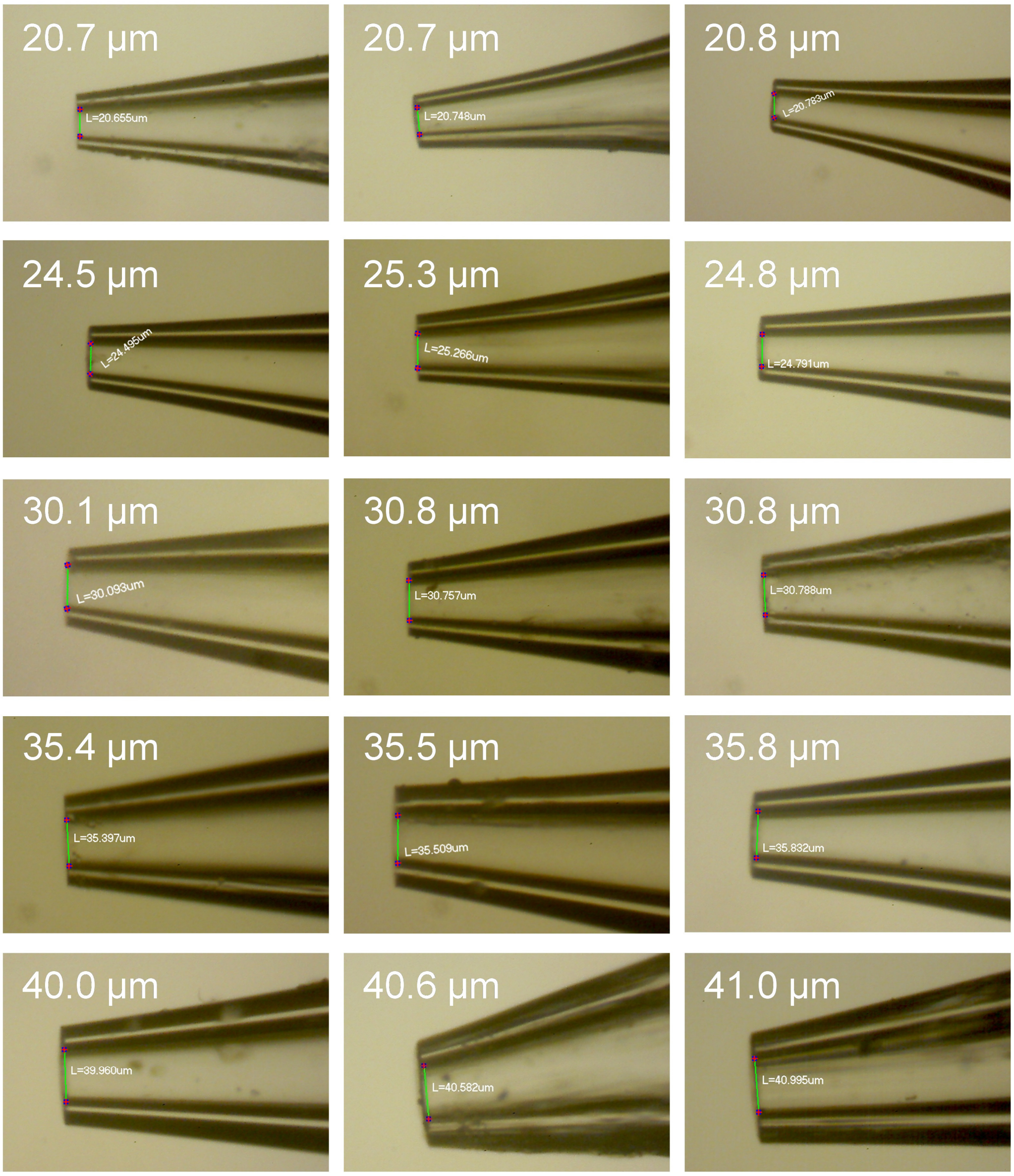


**Figure S9. Typical images of the capillary probe tips with different inner diameters (20, 25, 30, 35, 40 μm).** The errors of the tip inner diameters of the probes could be controlled in the range of ± 1 μm.
